# Comparison of Mortality and Major Cardiovascular Events Among Adults With Type 2 Diabetes Using Human vs Analogue Insulins

**DOI:** 10.1001/jamanetworkopen.2019.18554

**Published:** 2020-01-24

**Authors:** Romain Neugebauer, Emily B. Schroeder, Kristi Reynolds, Julie A. Schmittdiel, Linda Loes, Wendy Dyer, Jay R. Desai, Gabriela Vazquez-Benitez, P. Michael Ho, Jeff P. Anderson, Noel Pimentel, Patrick J. O’Connor

**Affiliations:** 1 Division of Research, Kaiser Permanente Northern California, Oakland; 2 Kaiser Permanente Colorado Institute for Health Research, Aurora; 3 Department of Research and Evaluation, Kaiser Permanente Southern California, Pasadena; 4 HealthPartners Institute, Minneapolis, Minnesota; 5 Rocky Mountain Regional Veterans Affairs and University of Colorado (Anschutz) Medical Center, Denver; 6 HealthPartners Center for Chronic Care Innovation, Minneapolis, Minnesota

## Abstract

**Importance:**

The comparative cardiovascular safety of analogue and human insulins in adults with type 2 diabetes who initiate insulin therapy in usual care settings has not been carefully evaluated using machine learning and other rigorous analytic methods.

**Objective:**

To examine the association of analogue vs human insulin use with mortality and major cardiovascular events.

**Design, Setting, and Participants:**

This retrospective cohort study included 127 600 adults aged 21 to 89 years with type 2 diabetes at 4 health care delivery systems who initiated insulin therapy from January 1, 2000, through December 31, 2013. Machine learning and rigorous inference methods with time-varying exposures were used to evaluate associations of continuous exposure to analogue vs human insulins with mortality and major cardiovascular events. Data were analyzed from September 1, 2017, through June 30, 2018.

**Exposures:**

On the index date (first insulin dispensing), participants were classified as using analogue insulin with or without human insulin or human insulin only.

**Main Outcomes and Measures:**

Overall mortality, mortality due to cardiovascular disease (CVD), myocardial infarction (MI), stroke or cerebrovascular accident (CVA), and hospitalization for congestive heart failure (CHF) were evaluated. Marginal structural modeling (MSM) with inverse probability weighting was used to compare event-free survival in separate per-protocol analyses. Adjusted and unadjusted hazard ratios and cumulative risk differences were based on logistic MSM parameterizations for counterfactual hazards. Propensity scores were estimated using a data-adaptive approach (machine learning) based on 3 nested covariate adjustment sets. Sensitivity analyses were conducted to address potential residual confounding from unmeasured differences in risk factors across delivery systems.

**Results:**

The 127 600 participants (mean [SD] age, 59.4 [12.6] years; 68 588 men [53.8%]; mean [SD] body mass index, 32.3 [7.1]) had a median follow-up of 4 quarters (interquartile range, 3-9 quarters) and experienced 5464 deaths overall (4.3%), 1729 MIs (1.4%), 1301 CVAs (1.0%), and 3082 CHF hospitalizations (2.4%). There were no differences in adjusted hazard ratios for continuous analogue vs human insulin exposure during 10 quarters for overall mortality (1.15; 95% CI, 0.97-1.34), CVD mortality (1.26; 95% CI, 0.86-1.66), MI (1.11; 95% CI, 0.77-1.45), CVA (1.30; 95% CI, 0.81-1.78), or CHF hospitalization (0.93; 95% CI, 0.75-1.11).

**Conclusions and Relevance:**

Insulin-naive adults with type 2 diabetes who initiate and continue treatment with human vs analogue insulins had similar observed rates of major cardiovascular events, CVD mortality, and overall mortality.

## Key Points

**Question:**

Are there significant differences in cardiovascular outcomes in adults with type 2 diabetes who use human insulin compared with those who use analogue insulin?

**Findings:**

In this cohort study of 127 600 adults with type 2 diabetes, no differences were found in overall mortality, cardiovascular mortality, myocardial infarction, stroke, and hospitalization for congestive heart failure.

**Meaning:**

In this study, insulin-naive adults with type 2 diabetes treated with human vs analogue insulin had similar rates of major cardiovascular events, mortality due to cardiovascular disease, and overall mortality.

## Teaser

This cohort study examines the association of analogue compared with human insulin use with mortality and major cardiovascular events among adults with type 2 diabetes.

## Introduction

Because major cardiovascular events and mortality due to cardiovascular disease (CVD) are the principal causes of excess mortality and health care costs in adults with type 2 diabetes, the selection of agents to lower glucose levels for those with type 2 diabetes is necessarily informed by the effects of various agents on major cardiovascular events and mortality, as well as by other factors such as rates of hypoglycemia, convenience of use, and medication costs.^1,2^ The US Food and Drug Administration has required randomized cardiovascular outcome trials for all agents to lower glucose levels approved since 2008. The results of these trials demonstrate that some agents confer substantive cardiovascular-related benefits among some individuals with type 2 diabetes, whereas other agents do not.^3,4,5,6,7,8,9^

Currently, approximately 90% of insulin users in the United States use analogue insulins, which were first introduced to the US market in 1996 and rapidly became widely used despite higher costs, because of effective marketing and studies indicating a lower rate of mild hypoglycemia.^1,10,11^ Although hypoglycemia rates related to various types of insulin have been extensively investigated, the effects of human compared with analogue insulins on cardiovascular events and mortality have received much less attention. Human insulin and the most commonly used analogue insulins were introduced before 2008 and thus were not evaluated in US Food and Drug Administration–mandated cardiovascular outcome trials. Some major clinical trials, such as the Diabetes Control and Complications Trial^12^ and United Kingdom Prospective Diabetes Study,^13^ did not include use of analogue insulins. Although more recent trials, such as ACCORD (Action to Control Cardiovascular Risk in Diabetes)^14^ and ADVANCE (Action in Diabetes and Vascular Disease: Preterax and Diamicron MR Controlled Evaluation),^15^ included the use of analogue insulins, the study designs preclude inferences about the cardiovascular safety of specific agents to lower glucose levels and direct comparison of the cardiovascular effects of human and analogue insulins.^12,15,16,17,18^

The price differential between human and analogue insulins and the lack of significant differences in rates of serious hypoglycemia in recent reports have sparked new interest in the use of human insulin as a way to make health care more affordable to patients with diabetes.^11,19^ However, a recent meta-analysis^20^ and a recent World Health Organization position paper on diabetes care^21,22^ note that few methodologically rigorous studies have compared the relative effect of human vs analogue insulins on rates of major cardiovascular events and mortality in adults with type 2 diabetes. Herein we report the results of a large, multisite, National Institutes of Health–funded retrospective cohort study designed to assess the occurrence of mortality, CVD mortality, acute myocardial infarction (MI), stroke or cerebrovascular accident (CVA), and hospitalization for congestive heart failure (CHF) in adults with type 2 diabetes who initiated and adhered to a regimen of human vs analogue insulin. The present study differs from prior investigations of this topic by including a large number of US participants receiving care in community-based clinics, having relatively complete clinical and clinical outcome data, and applying current guidelines for machine learning and other modern statistical techniques that accommodate time-varying exposures and large health care databases under explicit assumptions such as those identified in this report.^23,24,25,26,27,28,29,30^

## Methods

### Study Design, Study Sites, and Data Sources

This retrospective cohort study aimed to emulate^24^ a randomized experiment in which insulin-naive adults with type 2 diabetes would have been randomized at the time of dispensing the first insulin prescription to a continuous regimen of human insulin only (HI group) or analogue insulin with or without human insulin (AI group). The study sites included 4 integrated health care delivery systems from the Health Care Services Research Network: HealthPartners in Minnesota, Kaiser Permanente Colorado, Kaiser Permanente Northern California, and Kaiser Permanente Southern California.^31^ Health system electronic medical records, administrative claims data, 2010 census data, and mortality data were used to identify eligible participants, insulin type and use, demographics, clinical values, outcome variables, and covariates. The institutional review board of HealthPartners reviewed, approved, and monitored the progression of this study and approved our request to waive written informed consent for participants in this retrospective cohort study. This study followed the Strengthening the Reporting of Observational Studies in Epidemiology (STROBE) reporting guideline.

**Figure 1.**
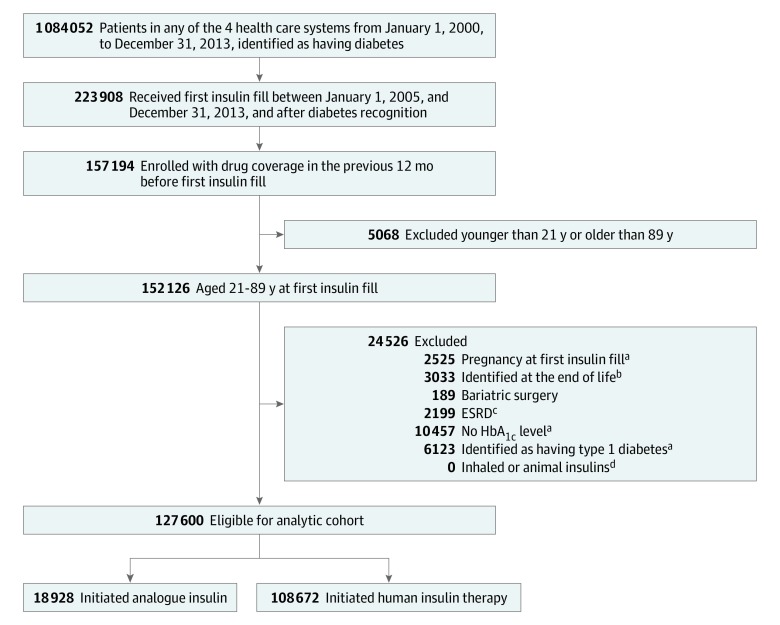
Flowchart of Participant Exclusions and Eligibility Data were extracted from the electronic health record and administrative databases using virtual data warehouse databases at each study site. Data from January 1, 2005, through December 31, 2013, are included. ESRD indicates end-stage renal disease; HbA_1c_, hemoglobin A_1c_. ^a^Identified based on diagnoses, procedure codes, or laboratory data in the 2 years before the index date, unless otherwise indicated. ^b^Indicates palliative care, hospice care, or stage IV cancer. ^c^Defined as estimated glomerular filtration rate of less than 15 mL/1.73 m^2^/min, dialysis, or transplant. ^d^Identified using pharmacy codes.

Overall, the combined membership of the 4 participating organizations was approximately 17 million members, of whom approximately 1.1 million individuals met criteria for diabetes from January 1, 2000, through December 31, 2013 (Figure 1).^32^ Operational definitions of type 2 diabetes and detailed definitions of other inclusion and exclusion criteria are presented in eMethods 1 in the Supplement.

### Participants

We searched the entire adult membership of the 4 participating Health Care Services Research Network health plans to identify those who filled a first insulin prescription from January 1, 2005, through December 31, 2013, and who met all eligibility criteria detailed in eMethods 1 in the Supplement on the date when insulin was first dispensed (index date). Following prospective study enrollment principles, participants were not excluded from the study based on information collected after the first insulin fill.

Participants were followed up from their index date until the earliest of (1) December 31, 2013 (administrative end of the study), (2) plan disenrollment (defined as a health insurance coverage gap of >90 days), or (3) death. For CVD mortality, the administrative end of study was December 31, 2011, owing to a 2-year lag of state death records. All participants with a first insulin prescription after that date were thus excluded from the analysis of CVD mortality.

### Exposures

Two exposure groups defined by continuous treatment with the same insulin therapy (HI and AI groups) were compared. In the primary analyses, participants were considered exposed to a given insulin therapy from its dispensing date until the earliest of 180 days after dispensing or the date of a new prescription fill. If a new insulin prescription was filled before the 180th day after a prior insulin prescription was dispensed, we assumed that the patient expended the prior insulin supply (ie, stockpiling of insulin was assumed to be null). In sensitivity analyses, continuous treatment with the same insulin therapy was determined based on the assumption that each prescription could last as long as 365 days instead of 180 days.

### Clinical Outcomes

Five clinical time-to-event outcomes were examined (eTable 1 in the Supplement). Acute MI (*International Statistical Classification of Diseases, Tenth Revision, Clinical Modification* [*ICD-9-CM*] code 410.xx), stroke/CVA (*ICD-9-CM* codes 430.xx, 431.xx, 433.x1, and 434.x1), and heart failure (*ICD-9-CM* codes 402.01, 402.11, 402.91, 404.01, 404.03, 404.11, 404.13, 404.91, 404.93, and 428.xx) were based on the inpatient principal discharge diagnosis. All-cause and CVD mortality were based on health system, state, and national vital statistics data. Mortality due to CVD included coronary heart disease, heart failure, cerebrovascular disease, peripheral artery disease, and atherosclerosis as defined by the primary cause of death.

### Covariates

Based on the current medical literature or consensus medical judgment, we identified a comprehensive list of covariates (eTable 2 and eTable 3 in the Supplement) potentially affecting the exposures, outcomes, and censoring events (plan disenrollment, adherence to the initial insulin regimen, and death). These included patient demographics, clinical values, comorbid conditions, concomitant medications, smoking, neighborhood-level socioeconomic variables, and clinician and site characteristics (eTables 4-6 in the Supplement).

### Statistical Analysis

Data were analyzed from September 1, 2017, through June 30, 2018. A separate analytic data set was constructed^33^ for each of the 5 clinical outcomes to conduct per-protocol analyses. Time-dependent variables (eMethods 2 in the Supplement) were updated every 90 days from the index date to the analytic end of follow-up, defined as the earliest of failure occurrence or one of the following right-censoring events: interruption of insulin therapy, switch in therapy type, start of pregnancy, or the administrative end of the study follow-up period.

To account for baseline confounding and time-dependent sources of bias from informative censoring,^34^ we used inverse probability weight (IPW) estimation to evaluate the counterfactual cumulative risks of failure if all participants were continuously exposed to human-only or analogue-containing insulin therapy.^23,35^ For each outcome, IPW was used to fit 2 logistic marginal structural models (MSM) for the discrete-time counterfactual hazards (eMethods 3 in the Supplement) during the first 2.5 years of follow-up: an MSM that relies on the proportionality assumption^27,36^ to provide a single summary effect measure estimate (hazard ratio) and a saturated MSM^29,37^ to provide estimates of differences in cumulative risks (at 1 and 2 years) between the 2 exposure regimens without reliance on the proportionality assumption.

Four approaches for estimating the propensity scores that define the IPW were considered. The first 3 approaches were based on logistic modeling (eMethods 4 in the Supplement) with different covariate adjustment sets^38,39^ (eMethods 5 in the Supplement). The fourth approach was based on data-adaptive propensity score estimation with a machine learning method known as Super Learning.^40^ Super Learning was used for adapting the covariate adjustment set that best predicts each propensity score outcome based on a logistic model (eMethods 6 in the Supplement).^41,42^ All IPWs were stabilized and truncated^43,44^ at 20. Adjusted effect measure estimates from the 2 MSMs and 4 propensity score estimation approaches considered were also compared with their unadjusted effect measure estimates.

Motivated by results from the evaluation of patterns of first insulin use across sites, we conducted post hoc site-specific sensitivity analyses restricted to data from the 3 sites that used similar proportions of human vs analogue insulin (sites 2-4) and data from the site (site 4) that had the most variability in the type of first insulin use during the years of study. Sensitivity analyses as well as the primary analyses include interaction terms between site and year of study entry variables in the propensity score logistic models used to predict the initial insulin therapy prescribed at the index date. Statistical details of the main and sensitivity analyses are provided in eMethods 7 in the Supplement. For each outcome analysis, we computed a 2-tailed *P* value for the statistical test that the area between the 2 survival curves is null (ie, the sum of the risk differences at each quarter is equal to 0). *P* < .05 indicated statistical significance.

## Results

Of the 1 084 052 participants with diabetes in the 4 health care systems, 223 908 had a first fill of insulin from January 1, 2005, through December 31, 2013. Of these, 127 600 participants with type 2 diabetes initiating insulin therapy were included in the main study cohort (mean [SD] age, 59.4 [12.6] years; 68 588 men [53.8%] and 59 012 women [46.2%]), with 108 672 (85.2%) in the HI group and 18 928 (14.8%) in the AI group (Figure 1). Only 95 300 of the 127 600 insulin-using participants were evaluated in the CVD mortality analyses for the reasons discussed earlier.

Table 1 describes selected demographic and clinical characteristics of participants at the index date in the main cohort by type of insulin initiated. The AI group was slightly younger (mean [SD] age, 58.8 [13.2] vs 59.5 [12.5] years) and had slightly higher rate of comorbidities (including coronary artery disease [18.7% vs 16.1%], CVA [2.2% vs 1.6%], and CHF [7.6% vs 6.5%]). Mean hemoglobin A_1c_ levels, blood pressure, and smoking rates were similar between the groups. Of the 127 600 patients, 98 965 (77.6%) initiated only long-acting insulin therapy at the index date, 10 379 (8.1%) initiated only short-acting insulin therapy, and 18 256 (14.3%) initiated a combination of both insulin types. Change in hemoglobin A_1c_ values after the index date and during the 2.5-year follow-up showed no systematic major differences between the AI and HI groups.

**Table 1.** Baseline Clinical and Demographic Characteristics of Participants^a^

Characteristic	HI Group (n = 108 672)^b^	AI Group (n = 18 928)^b^
Age, mean (SD), y^c^	59.5 (12.5)	58.8 (13.2)
Male sex	58 178 (53.5)	10 410 (55.0)
BMI, mean (SD)^c^	32.3 (7.0)	32.1 (7.2)
CABG surgery^d^	917 (0.8)	298 (1.6)
CAD^d^	17 488 (16.1)	3545 (18.7)
Coronary stent^d^	1712 (1.6)	410 (2.2)
Stroke event^d^	1791 (1.6)	416 (2.2)
CHF^d^	7050 (6.5)	1438 (7.6)
Hospitalization for CHF^d^	1757 (1.6)	465 (2.5)
Elixhauser comorbidity score, mean (SD)^e^	4.9 (2.5)	5.1 (2.8)
eGFR, mean (SD), mL/1.73 m^2^/min^c^	83.1 (31.0)	82.0 (31.7)
HbA_1c_ level, mean (SD), %^c^	9.5 (2.1)	9.4 (2.2)
Hypertension medications^c^	87 240 (80.3)	14 676 (77.5)
Hypertension diagnosis^d^	84 760 (78.0)	14 444 (76.3)
LDL cholesterol level, mg/dL^c^	92.1 (35.1)	95.6 (36.4)
Race/ethnicity		
Hispanic	33 683 (31.0)	5154 (27.2)
Black	12 506 (11.5)	2516 (13.3)
Hawaiian or Pacific Islander	1574 (1.4)	219 (1.2)
Asian	11 881 (10.9)	1771 (9.4)
Native American	590 (0.5)	117 (0.6)
White	45 060 (41.5)	8526 (45.0)
Missing	3378 (3.1)	625 (3.3)
Systolic BP, mean (SD), mm Hg^c^	128.5 (12.1)	128.3 (12.8)
Diastolic BP, mean (SD), mm Hg^c^	73.9 (8.3)	73.5 (8.6)
Smoking status^c^		
Current	15 838 (14.6)	2825 (14.9)
Never	53 453 (49.2)	9140 (48.3)
Former	39 381 (36.2)	6963 (36.8)

Abbreviations: AI, analogue insulin; BMI, body mass index (calculated as weight in kilograms divided by height in meters squared); BP, blood pressure; CABG, coronary artery bypass graft; CAD, coronary artery disease; CHF, congestive heart failure; eGFR, estimated glomerular filtration rate; HbA_1c_, hemoglobin A_1c_; HI, human insulin; LDL, low-density lipoprotein.

SI conversion factors: To convert HbA_1c_ level to proportion of hemoglobin, multiply by 0.01; LDL cholesterol to millimoles per liter, multiply by 0.0259.

^a^ Unless otherwise indicated, data are expressed as number (percentage) of patients. Percentages have been rounded and may not total 100.

^b^ The HI group includes patients receiving HI only; the AI group includes patients receiving AI with or without HI.

^c^ At index date or for most recent test performed before index date.

^d^ Based on 2 or more diagnosis codes or 1 or more procedure codes in the 2 years before the index date.

^e^ Calculated using the method of Elixhauser based on data from the 2-year period before the index date.

The median time from the index date to the analytic end of follow-up (eg, owing to a switch in insulin therapy) was 4 quarters in the main and CVD cohorts, with an interquartile range of 3 to 9 quarters in the main cohort and 3 to 8 quarters in the CVD cohort. We thus restricted the evaluation of hazards to the first 10 quarters of follow-up. Table 2 quantifies exposure time to analogue or human insulin and the number of events for each of the 5 outcomes. Overall, participants experienced 5464 deaths (4.3%), 1729 MIs (1.4%), 1301 CVAs (1.0%), and 3082 CHF hospitalizations (2.4%). Table 2 also displays distribution of reasons for end of follow-up in all primary analyses. Interruption of initial insulin therapy was the primary source of right censoring (57.1% for MI; 57.7% for mortality; 57.2% for CVA; 56.6% for CHF; and 54.6% for CVD mortality) and occurred for the following 4 reasons in order of decreasing frequency: a gap of more than 180 days between 2 consecutive insulin prescriptions dispensed, discontinuation of the initial insulin therapy with no subsequent insulin refill before the study end of follow-up, switching from analogue to human insulin therapy or vice versa, or dispensing of inhaled or animal insulins. For all primary analyses, more than half (>57%) of participants whose analytic end of follow-up was due to a gap in or discontinuation of the initial insulin therapy were right censored in the third quarter of follow-up because they did not refill a prescription for insulin within 180 days from the index date. This amounts to more than 26% of participants in the main and CVD cohorts being right censored owing to not filling a second insulin prescription within 180 days from their first insulin fill.

**Table 2.** Event Rates and Reasons for End of Analytic Follow-up

Reason for End of Analytic Follow-up	Study Events				
	MI (n = 127 600)	Mortality (n = 127 600)	CVA (n = 127 600)	CHF (n = 127 600)	CVD Mortality (n = 95 300)
Administrative end of follow-up, No. (%)	36 187 (28.4)	36 691 (28.8)	36 398 (28.5)	36 058 (28.3)	31 171 (32.7)
End enrollment in health or pharmacy insurance, No. (%)	11 442 (9.0)	11 429 (9.0)	11 476 (9.0)	11 387 (8.9)	7889 (8.3)
Start of pregnancy, No. (%)	361 (0.3)	362 (0.3)	361 (0.3)	360 (0.3)	268 (0.3)
Death as a right-censoring event, No. (%)	4983 (3.9)	NA	5043 (4.0)	4535 (3.6)	2328 (2.4)
Outcome, No. (%)	1729 (1.4)	5464 (4.3)	1301 (1.0)	3082 (2.4)	1588 (1.7)
Interruption of initial insulin therapy, No. (%)	72 898 (57.1)	73 654 (57.7)	73 021 (57.2)	72 178 (56.6)	52 056 (54.6)
Gap, No.	41 152	41 553	41 255	40 857	31 136
Switch, No.	13 044	13 232	13 086	12 902	9842
No fill, No.	18 698	18 865	18 676	18 415	11 074
Event rates by exposure, AI group/HI group^a^					
No. with outcome	269/1460	840/4624	222/1079	486/2596	257/1331
Person-time in quarters	112 806/651 262	114 556/662 288	113 055/654 135	112 070/647 937	87 604/416 283

Abbreviations: AI, analogue insulin; CHF, congestive heart failure; CVA, cerebrovascular accident; CVD, cardiovascular disease; HI, human insulin; MI, myocardial infarction; NA, not applicable.

^a^ The AI group includes patients receiving AI with or without HI; the HI group includes patients receiving HI only.

**Figure 2.**
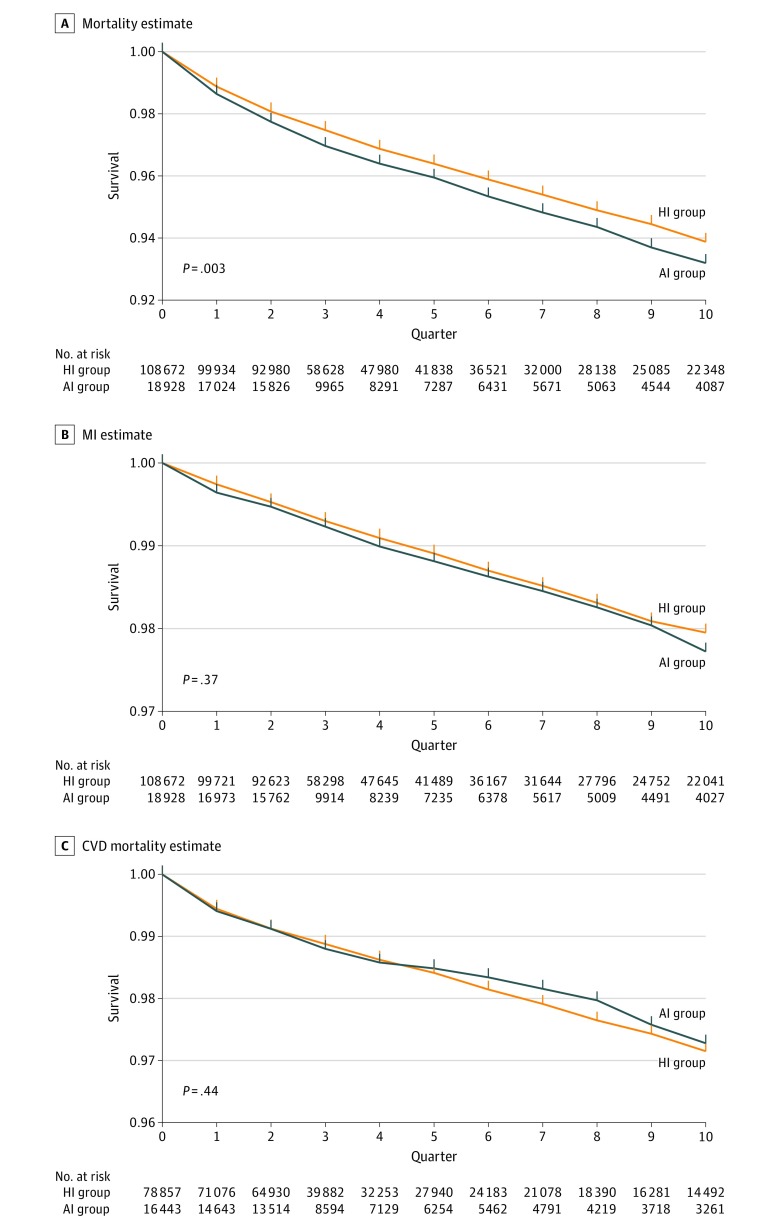
Crude Survival Curves for Mortality, Myocardial Infarction (MI), and Mortality Due to Cardiovascular Disease (CVD) Unadjusted estimates of the survival curves by insulin therapy regimen are shown for 3 of the 5 outcomes studied. The *P* value on each plot is the 2-tailed *P* value of the statistical test that the area between the 2 survival curves is null (ie, the sum of the risk differences at each quarter is equal to 0). AI group indicates patients continuously receiving analogue insulin with or without human insulin; HI group, patients continuously receiving human insulin only.

**Figure 3.**
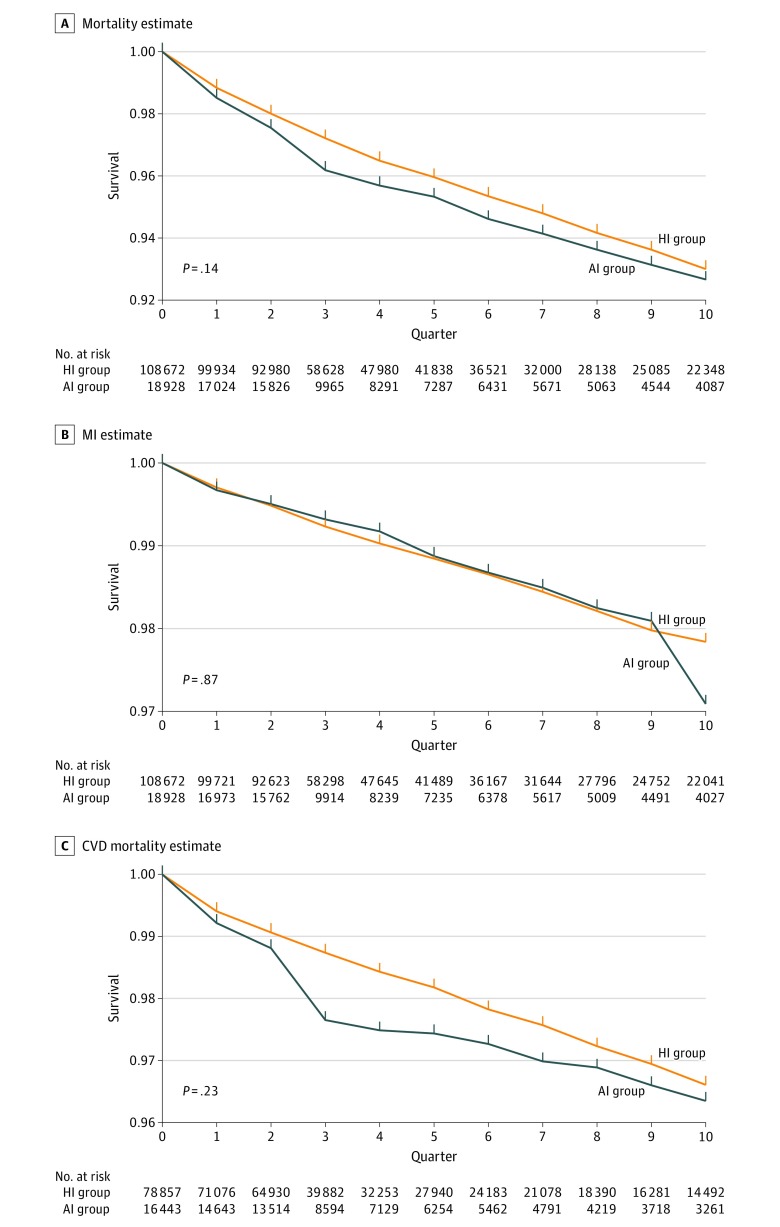
Adjusted Survival Curves for Mortality, Myocardial Infarction (MI), and Mortality Due to Cardiovascular Disease (CVD) Using Inverse Probability Weight Estimation to Fit a Saturated Marginal Structural Model Adjusted estimates of the survival curves by insulin therapy regimen are shown for 3 of the 5 outcomes studied. The *P *value on each plot is the 2-tailed *P* value of the statistical test that the area between the 2 survival curves is null (ie, the sum of the risk differences at each quarter is equal to 0). AI group indicates patients continuously receiving analogue insulin with or without human insulin; HI group, patients continuously receiving human insulin only.

Figure 2 displays the results of the unadjusted (crude) primary per-protocol analyses for mortality, MI, and CVD mortality and includes data on the number of participants at risk for each outcome in each quarter during the analysis period. Figure 3 displays the adjusted (IPW) primary per-protocol analyses based on the saturated MSM and Super Learning for propensity score estimation. Additional results and results for CHF and CVA are presented in eFigures 1 to 5 in the Supplement. Table 3 shows the adjusted hazard ratios and risk differences at 1 and 2 years along with their 95% CIs from the primary per-protocol analyses of each outcome using data-adaptive estimation of the propensity score. Adjusted estimates from the primary per-protocol analyses provide no evidence of a statistically significant difference in cumulative risks between the HI and AI groups during the first 10 quarters of follow-up for any outcome irrespective of the propensity score estimation approach used. Adjusted hazard ratios and 95% CIs for continuous analogue vs human insulin exposure also demonstrated no statistically significant associations with overall mortality (1.15; 95% CI, 0.97-1.34), CVD mortality (1.26; 95% CI, 0.86-1.66), MI (1.11; 95% CI, 0.77-1.45), CVA (1.30; 95% CI, 0.81-1.78), and CHF hospitalization (0.93; 95% CI, 0.75-1.11). Estimations of risk differences at 1 and 2 years (Table 3) were consistent with these results.

**Table 3.** Primary Analysis Results^a^

Outcome	HR (95% CI)	RD (95% CI) at 1 y	RD (95% CI) at 2 y
Overall mortality	1.15 (0.97 to 1.34)	0.008 (−0.001 to 0.017)	0.005 (−0.006 to 0.016)
MI	1.11 (0.77 to 1.45)	−0.002 (−0.004 to 0.001)	−0.0004 (−0.005 to 0.005)
Hospitalization for CHF	0.93 (0.75 to 1.11)	−0.002 (−0.006 to 0.003)	−0.005 (−0.011 to 0.001)
Stroke or CVA	1.30 (0.81 to 1.78)	0.004 (−0.002 to 0.009)	0.004 (−0.003 to 0.011)
CVD mortality	1.26 (0.86 to 1.66)	0.009 (−0.001 to 0.020)	0.003 (−0.008 to 0.014)

Abbreviations: CHF, congestive heart failure; CVA, cerebrovascular accident; CVD, cardiovascular disease; HR, hazard ratio; MI, myocardial infarction; RD, risk difference.

^a^ Data are given from the primary analysis for each outcome. The reference exposure regimen is continuous exposure to human insulin therapy.

eMethods 7 in the Supplement provides the results of all primary and sensitivity analyses based on the 2 MSM and 4 propensity score estimation approaches considered, including details of propensity score estimation (eTables 7-12 in the Supplement), patterns of first insulin use across sites (eTable 13 in the Supplement), and follow-up time by quarter (eTables 14-28 in the Supplement). Null findings from the primary per-protocol analyses (Figure 2, Figure 3, and eTables 29-33 in the Supplement) are generally supported by the adjusted estimates from sensitivity per protocol analyses (eFigures 1-5 in the Supplement). Inverse probability weights are provided in eTables 34 to 38 in the Supplement.

## Discussion

The results of this cohort study show no consistent statistically significant differences in rates of MI, CVA, CHF hospitalizations, CVD mortality, or overall mortality between adults with type 2 diabetes initiating human vs analogue insulin treatment. Unadjusted analyses showed some results that favored the HI over the AI groups, but analyses adjusted for possible baseline or time-varying confounding and informative right censoring using standard and machine learning estimates of propensity scores based on 3 covariate adjustment sets showed no consistent differences in outcomes across exposure groups.

To our knowledge, no randomized clinical trials have compared the relative effects of human and analogue insulin on major cardiovascular events or mortality.^20,45^ Prior cohort studies of the relative effects of human vs analogue insulin on major cardiovascular outcomes have reported mixed results, with most studies having significant limitations related to selection of participants, sample size, or description of analytic details.^45,46,47,48^ Several cohort studies focused exclusively on bolus insulin preparations, included relatively small numbers of participants, were limited to US veterans, or may have included in the analysis cardiovascular events that occurred before the initiation of insulin therapy.^45,47,49^

Our results address many of the limitations of prior studies, including the inclusion of bolus and basal preparations of analogue and human insulin, inclusion of a large number of eligible participants from multiple US health care systems, systematic ascertainment of cardiovascular events and CVD mortality, inclusion of a broad representation of adults with type 2 diabetes being treated predominantly in primary care settings, and use of sophisticated analytic methods including but not limited to machine learning. The validity of results with the per-protocol analyses based on IPW estimation used in this study relies on the usual assumption of no unmeasured confounding^35^ or sources of selection bias,^34^ that is, the sequential randomization assumption. Upholding this assumption relies on the selection of an adequate set of covariates for bias adjustment. Several alternate pragmatic criteria for covariate selection, including machine learning, have been proposed,^39,41,42,50,51,52^ and we selected 4 such alternatives herein to evaluate consistency of results across analytic methods.

The extensive sensitivity analyses conducted in this study (eMethods 7 in the Supplement) led to multiple comparisons when analyzing the association of human vs analogue insulin with 5 clinical outcomes. Although multiple comparisons do not change point estimates and corresponding (pointwise) CIs, there is no correction to our *P* values to compensate for multiple hypothesis testing. This observation further supports the main finding of no significant differences in mortality or major cardiovascular events between the AI and HI groups.

Our results suggest that cardiovascular outcomes and mortality should not be a motivating factor in the decision to start human vs analogue insulin therapy in insulin-naive adults with type 2 diabetes. Other relevant factors to consider include hypoglycemia, glycemic control, cost, and ease of use. Recent reports have shown similar effects of human and analogue insulins on control of glucose levels^53,54^ and serious hypoglycemic events in primary care practice,^11^ which suggest that human and analogue insulins are safe and effective treatments in type 2 diabetes.^11,19,53,54,55,56^ The wholesale acquisition cost or list price of a 10-mL vial of human insulin was $24.90 for short- or intermediate-acting insulin, compared with $283 for a 10-mL vial of long-acting analogue insulin glargine and $289 for a 10-mL vial of rapid-acting insulin aspart analogue, according to data published in May 2019.^57^

### Limitations

Several factors constrain the interpretation of our results. First, even with the use of rigorous statistical methods and a large sample size, the cohort study design is a limitation. However, owing to the high cost of conducting large randomized trials in a rapidly evolving insulin market, there is little chance that a large randomized trial will address cardiovascular outcomes of human vs other insulins, although manufacturers have compared newer and older analogue insulins. Results show few differences in cardiovascular events, suggesting that newer analogue insulins are unlikely to have better cardiovascular outcomes than the analogue insulins we evaluated.^58,59^ Second, human insulin was the initial treatment for 5.0%, 82.5%, 90.4%, and 96.1% of participants at our 4 study sites owing to formulary preferences. We exploited temporal variability in prescription rates at one of the sites in a sensitivity analysis to address potential concerns over residual bias from unobserved differences in risk factors between human and analogue insulin users at the other 3 sites that consistently favored 1 insulin type during the years of the study. Third, we elected to include short-acting and long-acting analogue and human insulin preparations in this analysis because the chief clinical choice patients and physicians make on a daily basis is between analogue and human insulin. Additional research to compare the cardiovascular safety of short- and rapid-acting vs long-acting insulins is warranted. It is challenging to precisely detect interruption in insulin exposure solely from pharmacy-dispensing data. To address this concern, we demonstrated that inferences were not sensitive to our assumption about the maximum duration of each insulin prescription (ie, 180 vs 365 days).

## Conclusions

This detailed analysis of a large data set using rigorous modern statistical methods and machine learning suggests that adults with type 2 diabetes who are new users of human or analogue insulin have similar rates of mortality, CVD mortality, and major cardiovascular events during 2.5 years of follow-up. These results suggest that cardiovascular outcomes and mortality should not be a motivating factor in the decision to start human vs analogue insulin therapy in insulin-naive adults with type 2 diabetes. Other relevant factors to consider include hypoglycemia, glycemic control, cost, and ease of use. These results contribute important new clinical information that can help inform insulin-related treatment decisions made by adults with type 2 diabetes and their clinicians.

## Notes

Supplement. **eMethods 1.** Cohort Construction **eMethods 2.** Data Structure and Notation **eMethods 3.** Causal Estimands and Inverse Probability Estimator **eMethods 4.** Denominator of the Inverse Probability Weights **eMethods 5.** Standard Propensity Score Estimation With 3 Covariate Adjustment Sets **eMethods 6.** Data-Adaptive Propensity Score Estimation **eMethods 7.** Results **eTable 1.** Sources of Data and Codes Used to Ascertain Major Cardiovascular Events and Mortality **eTable 2.** Part I of II: Brief Description of All Attributes (L) in the Covariate Adjustment Sets **eTable 3.** Part II of II: Brief Description of All Attributes (L) in the Covariate Adjustment Sets **eTable 4.** Part I of II: List of Covariates Considered in the Various Analyses and Whether They Are Assumed to Impact Exposure Decisions, Censoring Events, or Outcomes **eTable 5.** Part II of II: List of Covariates Considered in the Various Analyses and Whether They Are Assumed to Impact Exposure Decisions, Censoring Events, or Outcomes **eTable 6.** Cutoffs Used to Discretize Continuous Covariates **eTable 7.** PS Estimation Approach 1 in AMI Primary Analysis **eTable 8.** Part I of II: PS Estimation Approach 2 in AMI Primary Analysis **eTable 9.** Part II of II: PS Estimation Approach 2 in AMI Primary Analysis **eTable 10.** Part I of II: PS Estimation Approach 3 in AMI Primary Analysis **eTable 11.** Part II of II: PS Estimation Approach 3 in AMI Primary Analysis **eTable 12.** PS Estimation Approach 4 in AMI Primary Analysis **eTable 13.** Distribution of Type of Initial Insulin Therapy for Patients in the Main Cohort by Site and Year of Cohort Entry **eTable 14.** Distribution of Follow-up Time (Expressed in 90-Day Intervals) for Patients Continuously Exposed to Analogue-Containing Insulin Therapy in the Primary AMI Analyses (All Sites Combined) **eTable 15.** Distribution of Follow-up Time (Expressed in 90-Day Intervals) for Patients Continuously Exposed to Human-Only Insulin Therapy in the Primary AMI Analyses (All Sites Combined) **eTable 16.** Summary Statistics of the Distribution of Follow-up Time (Expressed in 90-Day Intervals) by Exposure Regimen in the Primary AMI Analyses (All Sites Combined) **eTable 17.** Distribution of Follow-up Time (Expressed in 90-Day Intervals) for Patients Continuously Exposed to Analogue-Containing Insulin Therapy in the Primary CHF Analyses (All Sites Combined) **eTable 18.** Distribution of Follow-up Time (Expressed in 90-Day Intervals) for Patients Continuously Exposed to Human-Only Insulin Therapy in the Primary CHF Analyses (All Sites Combined) **eTable 19.** Summary Statistics of the Distribution of Follow-up Time (Expressed in 90-Day Intervals) by Exposure Regimen in the Primary CHF Analyses (All Sites Combined) **eTable 20.** Distribution of Follow-up Time (Expressed in 90-Day Intervals) for Patients Continuously Exposed to Analogue-Containing Insulin Therapy in the Primary CVA Analyses (All Sites Combined) **eTable 21.** Distribution of Follow-up Time (Expressed in 90-Day Intervals) for Patients Continuously Exposed to Human-Only Insulin Therapy in the Primary CVA Analyses (All Sites Combined) **eTable 22.** Summary Statistics of the Distribution of Follow-up Time (Expressed in 90-Day Intervals) by Exposure Regimen in the Primary CVA Analyses (All Sites Combined) **eTable 23.** Distribution of Follow-up Time (Expressed in 90-Day Intervals) for Patients Continuously Exposed to Analogue-Containing Insulin Therapy in the Primary CVD Mortality Analyses (All Sites Combined) **eTable 24.** Distribution of Follow-up Time (Expressed in 90-Day Intervals) for Patients Continuously Exposed to Human-Only Insulin Therapy in the Primary CVD Mortality Analyses (All Sites Combined) **eTable 25.** Summary Statistics of the Distribution of Follow-up Time (Expressed in 90-Day Intervals) by Exposure Regimen in the Primary CVD Mortality Analyses (All Sites Combined) **eTable 26.** Distribution of Follow-up Time (Expressed in 90-Day Intervals) for Patients Continuously Exposed to Analogue-Containing Insulin Therapy in the Primary All-Cause Mortality Analyses (All Sites Combined) **eTable 27.** Distribution of Follow-up Time (Expressed in 90-Day Intervals) for Patients Continuously Exposed to Human-Only Insulin Therapy in the Primary All-Cause Mortality Analyses (All Sites Combined) **eTable 28.** Summary Statistics of the Distribution of Follow-up Time (Expressed in 90-Day Intervals) by Exposure Regimen in the Primary All-Cause Mortality Analyses (All Sites Combined) **eTable 29.** AMI Results **eTable 30.** CHF Results **eTable 31.** CVA Results **eTable 32.** CVD Mortality Results **eTable 33.** All-Cause Mortality Results **eTable 34.** Summary Statistics of the Inverse Probability Weights (IPW) Involved in the AMI Analyses **eTable 35.** Summary Statistics of the Inverse Probability Weights (IPW) Involved in the CHF Analyses **eTable 36.** Summary Statistics of the Inverse Probability Weights (IPW) Involved in the CVA Analyses **eTable 37.** Summary Statistics of the Inverse Probability Weights (IPW) Involved in the CVD Mortality Analyses **eTable 38.** Summary Statistics of the Inverse Probability Weights (IPW) Involved in the All-Cause Mortality Analyses **eFigure 1.** Survival Curve Estimates for AMI (Primary and Sensitivity Analyses) **eFigure 2.** Survival Curve Estimates for CHF (Primary and Sensitivity Analyses) **eFigure 3.** Survival Curve Estimates for CVA (Primary and Sensitivity Analyses) **eFigure 4.** Survival Curve Estimates for CVD Mortality (Primary and Sensitivity Analyses) **eFigure 5.** Survival Curve Estimates for All-Cause Mortality (Primary and Sensitivity Analyses) **eReferences.**
