# Supplementary material for: Comparison of Mortality and Major Cardiovascular Events Among Adults With Type 2 Diabetes Using Human vs Analogue Insulins
Source: JAMA Netw Open. 2020 Jan 24;3(1):e1918554. doi: 10.1001/jamanetworkopen.2019.18554 (PMC6991251; doi:10.1001/jamanetworkopen.2019.18554)
Supplement: Supplement. — eMethods 1. Cohort Construction eMethods 2. Data Structure and Notation eMethods 3. Causal Estimands and Inverse Probability Estimator eMethods 4. Denominator of the Inverse Probability Weights eMethods 5. Standard Propensity Score Estimation With 3 Covariate Adjustment Sets eMethods 6. Data-Adaptive Propensity Score Estimation eMethods 7. Results eTable 1. Sources of Data and Codes Used to Ascertain Major Cardiovascular Events and Mortality eTable 2. Part I of II: Brief Description of All Attributes (L) in the Covariate Adjustment Sets eTable 3. Part II of II: Brief Description of All Attributes (L) in the Covariate Adjustment Sets eTable 4. Part I of II: List of Covariates Considered in the Various Analyses and Whether They Are Assumed to Impact Exposure Decisions, Censoring Events, or Outcomes eTable 5. Part II of II: List of Covariates Considered in the Various Analyses and Whether They Are Assumed to Impact Exposure Decisions, Censoring Events, or Outcomes eTable 6. Cutoffs Used to Discretize Continuous Covariates eTable 7. PS Estimation Approach 1 in AMI Primary Analysis eTable 8. Part I of II: PS Estimation Approach 2 in AMI Primary Analysis eTable 9. Part II of II: PS Estimation Approach 2 in AMI Primary Analysis eTable 10. Part I of II: PS Estimation Approach 3 in AMI Primary Analysis eTable 11. Part II of II: PS Estimation Approach 3 in AMI Primary Analysis eTable 12. PS Estimation Approach 4 in AMI Primary Analysis eTable 13. Distribution of Type of Initial Insulin Therapy for Patients in the Main Cohort by Site and Year of Cohort Entry eTable 14. Distribution of Follow-up Time (Expressed in 90-Day Intervals) for Patients Continuously Exposed to Analogue-Containing Insulin Therapy in the Primary AMI Analyses (All Sites Combined) eTable 15. Distribution of Follow-up Time (Expressed in 90-Day Intervals) for Patients Continuously Exposed to Human-Only Insulin Therapy in the Primary AMI Analyses (All Sites Combined) eTable 16. Summary Statistics of the Distri [file jamanetwopen-3-e1918554-s001.pdf]

Supplementary Online Content

Neugebauer R, Schroeder EB, Reynolds K, et al. Comparison of mortality and major cardiovascular events among adults with type 2 diabetes using human vs analogue insulins. *JAMA Netw Open.* 2020;3(1):e1918554. doi:10.1001/jamanetworkopen.2019.18554

eMethods 1. Cohort Construction

eMethods 2. Data Structure and Notation

eMethods 3. Causal Estimands and Inverse Probability Estimator

eMethods 4. Denominator of the Inverse Probability Weights

eMethods 5. Standard Propensity Score Estimation With 3 Covariate Adjustment Sets

eMethods 6. Data-Adaptive Propensity Score Estimation

eMethods 7. Results

eTable 1. Sources of Data and Codes Used to Ascertain Major Cardiovascular Events and Mortality

eTable 2. Part I of II: Brief Description of All Attributes (L) in the Covariate Adjustment Sets

eTable 3. Part II of II: Brief Description of All Attributes (L) in the Covariate Adjustment Sets

eTable 4. Part I of II: List of Covariates Considered in the Various Analyses and Whether They Are Assumed to Impact Exposure Decisions, Censoring Events, or Outcomes

eTable 5. Part II of II: List of Covariates Considered in the Various Analyses and Whether They Are Assumed to Impact Exposure Decisions, Censoring Events, or Outcomes

eTable 6. Cutoffs Used to Discretize Continuous Covariates

eTable 7. PS Estimation Approach 1 in AMI Primary Analysis

eTable 8. Part I of II: PS Estimation Approach 2 in AMI Primary Analysis

eTable 9. Part II of II: PS Estimation Approach 2 in AMI Primary Analysis

eTable 10. Part I of II: PS Estimation Approach 3 in AMI Primary Analysis

eTable 11. Part II of II: PS Estimation Approach 3 in AMI Primary Analysis

eTable 12. PS Estimation Approach 4 in AMI Primary Analysis

© 2020 Neugebauer R et al. *JAMA Network Open.*

eTable 13. Distribution of Type of Initial Insulin Therapy for Patients in the Main Cohort by Site and Year of Cohort Entry

eTable 14. Distribution of Follow-up Time (Expressed in 90-Day Intervals) for Patients Continuously Exposed to Analogue-Containing Insulin Therapy in the Primary AMI Analyses (All Sites Combined)

eTable 15. Distribution of Follow-up Time (Expressed in 90-Day Intervals) for Patients Continuously Exposed to Human-Only Insulin Therapy in the Primary AMI Analyses (All Sites Combined)

eTable 16. Summary Statistics of the Distribution of Follow-up Time (Expressed in 90-Day Intervals) by Exposure Regimen in the Primary AMI Analyses (All Sites Combined)

eTable 17. Distribution of Follow-up Time (Expressed in 90-Day Intervals) for Patients Continuously Exposed to Analogue-Containing Insulin Therapy in the Primary CHF Analyses (All Sites Combined)

eTable 18. Distribution of Follow-up Time (Expressed in 90-Day Intervals) for Patients Continuously Exposed to Human-Only Insulin Therapy in the Primary CHF Analyses (All Sites Combined)

eTable 19. Summary Statistics of the Distribution of Follow-up Time (Expressed in 90-Day Intervals) by Exposure Regimen in the Primary CHF Analyses (All Sites Combined)

eTable 20. Distribution of Follow-up Time (Expressed in 90-Day Intervals) for Patients Continuously Exposed to Analogue-Containing Insulin Therapy in the Primary CVA Analyses (All Sites Combined)

eTable 21. Distribution of Follow-up Time (Expressed in 90-Day Intervals) for Patients Continuously Exposed to Human-Only Insulin Therapy in the Primary CVA Analyses (All Sites Combined)

eTable 22. Summary Statistics of the Distribution of Follow-up Time (Expressed in 90-Day Intervals) by Exposure Regimen in the Primary CVA Analyses (All Sites Combined)

eTable 23. Distribution of Follow-up Time (Expressed in 90-Day Intervals) for Patients Continuously Exposed to Analogue-Containing Insulin Therapy in the Primary CVD Mortality Analyses (All Sites Combined)

eTable 24. Distribution of Follow-up Time (Expressed in 90-Day Intervals) for Patients Continuously Exposed to Human-Only Insulin Therapy in the Primary CVD Mortality Analyses (All Sites Combined)

eTable 25. Summary Statistics of the Distribution of Follow-up Time (Expressed in 90-Day Intervals) by Exposure Regimen in the Primary CVD Mortality Analyses (All Sites Combined)

eTable 26. Distribution of Follow-up Time (Expressed in 90-Day Intervals) for Patients Continuously Exposed to Analogue-Containing Insulin Therapy in the Primary All-Cause Mortality Analyses (All Sites Combined)

eTable 27. Distribution of Follow-up Time (Expressed in 90-Day Intervals) for Patients Continuously Exposed to Human-Only Insulin Therapy in the Primary All-Cause Mortality Analyses (All Sites Combined)

eTable 28. Summary Statistics of the Distribution of Follow-up Time (Expressed in 90-Day Intervals) by Exposure Regimen in the Primary All-Cause Mortality Analyses (All Sites Combined)

eTable 29. AMI Results

eTable 30. CHF Results

eTable 31. CVA Results

eTable 32. CVD Mortality Results

eTable 33. All-Cause Mortality Results

eTable 34. Summary Statistics of the Inverse Probability Weights (IPW) Involved in the AMI Analyses

eTable 35. Summary Statistics of the Inverse Probability Weights (IPW) Involved in the CHF Analyses

eTable 36. Summary Statistics of the Inverse Probability Weights (IPW) Involved in the CVA Analyses

eTable 37. Summary Statistics of the Inverse Probability Weights (IPW) Involved in the CVD Mortality Analyses

eTable 38. Summary Statistics of the Inverse Probability Weights (IPW) Involved in the All-Cause Mortality Analyses

eFigure 1. Survival Curve Estimates for AMI (Primary and Sensitivity Analyses)

eFigure 2. Survival Curve Estimates for CHF (Primary and Sensitivity Analyses)

eFigure 3. Survival Curve Estimates for CVA (Primary and Sensitivity Analyses)

eFigure 4. Survival Curve Estimates for CVD Mortality (Primary and Sensitivity Analyses)

eFigure 5. Survival Curve Estimates for All-Cause Mortality (Primary and Sensitivity Analyses)

eReferences.

This supplementary material has been provided by the authors to give readers additional information about their work.

eMethods 1 - Cohort Construction

We searched the electronic health records and administrative databases of the HP, KPCO, KPNC, KPSC health plans to identify all diabetes patients between 1/1/2000 and 12/31/2013 with a first insulin dispensing between 1/1/2005 and 12/31/2013. The algorithm used to identify diabetes patients is described below (second bullet). The date of first insulin dispensing is referred to as the index date. Each patient who met all of the following criteria was included in the main study cohort:

- age on index date $\geq$21 and $\leq$89
- diabetes recognition occurred before or on index date where the diabetes recognition date was defined from the patient's diagnoses from inpatient, ambulatory, laboratory, and pharmacy encounters. Specifically, diabetes recognition was defined as the earlier of one inpatient diagnosis (ICD-9-CM 250.x, 357.2, 366.41, 362.01-362.07) or any combination of two of the following events occurring within a 24-month period of time, using the date of the first event in the pair as the identification date: 1) A1C > 6.5% (48 mmol/mol); 2) fasting plasma glucose > 126 mg/dl (7.0 mmol/L); 3) random plasma glucose > 200 mg/dl (11.1 mmol/L); 4) an outpatient diagnosis code (same codes as inpatient); 5) any anti-hyperglycemic medication dispense. For example, an individual with an A1C of 7.5% (57 mmol/mol) followed by an outpatient diagnosis of diabetes would be identified with diabetes on the (earlier) date of the A1C, with a laboratory result as the primary source. When the two events used for identification came from the same source (e.g., two outpatient diagnoses), they were required to occur on separate dates, but no more than 24-months apart. Note the following exception: two dispensings of metformin, thiazolidinediones, or liraglutide – with no other indication of diabetes – was not counted because these agents could be used for diabetes prevention, weight loss or to treat polycystic ovarian syndrome. Events that were identified during a pregnancy (within 270 days prior to a delivery) were excluded from consideration
- minimum of 12 months of health plan enrollment before index date and allowing for multiple gaps not exceeding 90 days combined
- minimum of 12 months of drug coverage before index date and allowing for multiple gaps not exceeding 90 days combined
- not pregnant on index date
- no evidence of bariatric surgery in the 2 years before the index date, i.e., no record of the following ICD-9 procedure and CPT-4 codes: 43.89, 44.31, 44.38, 44.39, 44.68, 44.69, 44.95 ; 43633, 43644, 43645, 43659, 43770, 43775, 43842, 43843, 43844, 43845, 43846, 43847
- no evidence of end stage renal disease in the 2 years before the index date, i.e., no record of the following ICD-9 diagnosis, ICD-9 procedure, and CPT-4 codes (kidney transplant): v42.0, 996.81 ; 55.6, 55.61, 55.69 ; 50360, 50365, 50380 and most recent GFR laboratory result (if any) $\geq$15 and no record of 2 or more of the following ICD-9 diagnosis, ICD-9 procedure, and CPT-4 codes dated >90 days apart as primary or secondary diagnosis (dialysis): 585.6, 458.21, v45.1, v45.11, v56, v56.x, v56.2, v56.8 ; 39.95, 54.98 ; 90921, 90925, 90935-90999
- no evidence of a stage 4 cancer diagnosis in the 2 years before the index date, i.e., no record of the following ICD-9 diagnosis codes 197.x, 198.x, 199.x
- no evidence of hospice or palliative care in the 2 years before the index date, i.e., no record of an hospice encounter and no record of the ICD-9 diagnosis code v66.7 and no record of the CPT code 99377 and 99378
- at least one A1c laboratory measurement recorded in the 2 years before the index date
- insulins dispensed on the index date do not include animal or inhaled insulins
- diabetes of type 2 defined by the following ratio being strictly lower than 50%: the number of ICD-9 diagnosis codes 250.x1 and 250.x3 (type 1) in the 2 years before the index date divided by the sum of this number and the number of ICD-9 codes 250.x0 and 250.x2 (type 2) in the 2 years before the index date. If this ratio is not defined (i.e., denominator is 0), the diabetes type is unknown and the patient excluded from the study cohort.

© 2020 Neugebauer R et al. *JAMA Network Open.*

In addition to these criteria above, KPCO patients living outside the Denver/Boulder area were excluded due to incomplete data capture.

eMethods 2 - Data Structure and Notation

All analyses in this report are based on analytic datasets constructed with the `MSMstructure` SAS macro[1] to coarsen daily EHR data using the 90-day unit of time, i.e., time-dependent variables are updated every 90 days in the resulting analytic datasets. More specifically, for each of the five failure time outcomes considered (eTable 1), a separate analytic dataset is constructed by collecting the realizations of the random variables described below for all patients in the main or CVD study cohort.

Follow-up time (expressed in 90-day units) is denoted by t and, by convention, the first 90 days of follow-up are denoted by $t = 0$. The time when the patient's follow-up ends is denoted by $\tilde{T}$ and is defined as the earliest of the time to failure denoted by T or the time to a right-censoring event denoted by C. When a patient is right-censored, i.e., $C < T$, the type of right-censoring event experienced by the patient is recorded and denoted by Γ with possible values 1-7 to represent the administrative end of study, disenrollment from the health plan, start of a pregnancy, switch in therapy type (i.e., crossover from human-only to analog-containing therapy or vice versa), initiation of a non standard insulin (i.e., inhaled or animal insulin), interruption of insulin therapy, or death, respectively. The indicator that the end of follow-up is due to the occurrence of a failure event is denoted by $\Delta = I(T \leq C)$, i.e., $\Delta = 1$ implies that $\tilde{T} = T$ and $\Delta = 0$ implies that $\tilde{T} = C$. The indicator that the patient initiated analog-containing insulin therapy on the index date is represented by the binary variable $A_1(0)$ (i.e., $A_1(0) = 0$ indicates exposure to human-only insulin therapy). The indicator of the patient's right-censored status at time t is denoted by $A_2(t)$. We thus have $A_2(t) = 0$ for $t = 0, \ldots, \tilde{T} - 1$ when $\tilde{T} \geq 1$ and $A_2(\tilde{T}) = 1 - \Delta$. The exposure variable denoted by $A(t)$ is defined by $A(0) = (A_1(0), A_2(0))$ and $A(t) = A_2(t)$ for $t > 0$. At each time point $t = 0, \ldots, \tilde{T}$, covariates such as A1c measurements (eTables 2-3) are denoted by a component $L_j(t)$ of the random vector $L(t)$ and defined from measurements that occur before the exposure at time t, $A(t)$, or are otherwise assumed not to be affected by the exposures at time t or thereafter, $(A(t), A(t+1), \ldots)$. If no such measurements were collected, each variable $L_j(t)$ is defined by convention using last observed value carried forward at $t > 0$. If no baseline measurements were collected for a continuous variable in $L(0)$, the variable is defined by convention as the median of the baseline values from patients with observed measurements at $t = 0$. For categorical variables in $L(0)$, a separate level is defined to encode missing baseline measurements. For each time-independent or time-dependent covariate L_j with at least one missing measurement (at baseline or at $t > 0$), an indicator of missing covariate measurement at time t is created and included as a distinct variable (e.g., to encode intensity of clinical monitoring) in the random vector $L(t)$ for all time points t. In addition, the vector of covariates $L(t)$ at time t include an outcome measurement denoted by $Y(t)$, i.e., $Y(t) \in L(t)$ for $t = 0, \ldots, \tilde{T}$. For each time point $t = 1, \ldots, \tilde{T} + 1$, the outcome is the indicator of past failure, i.e., $Y(t) = I(T \leq t - 1)$ and $Y(0) = 0$ by convention. By definition, the outcome is thus 0 for $t = 0, \ldots, \tilde{T}$, not observed at $t = \tilde{T} + 1$ if $\Delta = 0$ and, 1 at $t = \tilde{T} + 1$ if $\Delta = 1$.

In short, the observed data in each analytic dataset are realizations of n copies O_i of the random process $O = \big(\tilde{T}, \Delta, (1-\Delta)\Gamma, \bar{L}(\tilde{T}), \bar{A}(\tilde{T}), \Delta Y(\tilde{T}+1)\big)$ where $n = 127,600$ in each of the four analytic datasets to evaluate AMI, CHF, CVA, all-cause mortality and $n = 95,300$ in the analytic dataset to evaluate CVD mortality. In the analyses of each dataset, we assumed[2] that the random variables O_i are independent and identically distributed.

To simplify expressions below, we use the overbar notation $\bar{\cdot}$ to denote the history of a variable $\cdot$ from baseline to time t (e.g., $\bar{A}(t) = (A(0), \ldots, A(t))$) and, by convention, $L(t)$ and $A(t)$ are nil when $t < 0$.

eMethods 3 - Causal Estimands and Inverse Probability Estimator

The following two working[3] logistic marginal structural models (MSMs) for discrete-time counterfactual hazards, $P(Y_{\bar{a}(t)}(t+1) = 1 \mid Y_{\bar{a}(t-1)}(t) = 0)$, were considered:

- a simple MSM whose parameterization mimics a common modeling practice that assumes constant hazard ratios over time (i.e., a model based on the proportionality assumption):

$$m_1(t, a_1(0) \mid \beta) = \left(1 + \exp\left(-\Big(\beta^0 I\big(a_1(0) = 1\big) + \sum_{j=1}^{10} \beta^j I\big(t = j-1\big)\Big)\right)\right)^{-1}$$

© 2020 Neugebauer R et al. *JAMA Network Open.*

- a saturated MSM whose parameterization permits hazard ratios to change over time:

$$m_2(t, a_1(0) \mid \beta) = \left(1 + \exp\left(-\Big(\sum_{j=1}^{10}\sum_{k=0}^{1} \beta^{j,k} I\big(t = j-1, a_1(0) = k\big)\Big)\right)\right)^{-1}$$

for $t = 0, \ldots, 9$ and $\bar{a}(t) = \bar{a}^0(t), \bar{a}^1(t)$ where, for each MSM, the collection of its coefficients is denoted by β and where $\bar{a}^0(t) = ((0,0), 0, \ldots, 0)$ represents continuous exposure to human-only insulin therapy and $\bar{a}^1(t) = ((1,0), 0, \ldots, 0)$ represents continuous exposure to analog-containing insulin therapy.

The standard[2,4] bounded and stabilized IPW estimator approach to fit each MSM was implemented in this report with the following choice of numerators (stabilizing factor) for the IP weights assigned to the person-time outcomes contributing to the weighted regression: $\prod_{j=0}^{t} P_n\big(A(j) = a^k(j) \mid \bar{A}(j-1) = \bar{a}^k(j-1)\big)$ with $k = 0, 1$ and $t = 0, \ldots, 9$ where each factor P_n denotes a sample mean. The resulting IPW estimator of the MSM coefficient β is denoted by β_n and define the various effect measures reported below.

The first MSM fit provided a single effect measure estimate $\exp(\beta_n^0)$ corresponding with an estimate of the constant causal hazard ratio (HR) $P(Y_{\bar{a}^1(t)}(t+1) = 1)/P(Y_{\bar{a}^0(t)}(t+1) = 1)$ under the proportionality assumption and rare event assumption. The second MSM fit was mapped into estimates of the counterfactual cumulative risks $P(Y_{\bar{a}^k(t)}(t+1) = 1)$ (equivalently, the counterfactual survival probability $P(T_{\bar{a}^k(t)} > t) = 1 - P(Y_{\bar{a}^k(t)}(t+1) = 1)$) as follows for $t = 0, \ldots, 9$ and $k = 0, 1$:

$$P_n(Y_{\bar{a}^k(t)}(t+1) = 1) \;=\; 1 - \prod_{j=0}^{t}\Big(1 - m_2(j, k \mid \beta_n)\Big).$$

These estimates of counterfactual cumulative risks defined three effect measure estimates:

- the difference between the areas under the two discrete-time survival curves (AUC):

$$\sum_{j=0}^{9}\Big(P_n(Y_{\bar{a}^1(j)}(j+1) = 1) - P_n(Y_{\bar{a}^0(j)}(j+1) = 1)\Big)$$

- the risk difference (RD) at 1 year: $P_n(Y_{\bar{a}^1(3)}(4) = 1) - P_n(Y_{\bar{a}^0(3)}(4) = 1)$
- the risk difference (RD) at 2 years: $P_n(Y_{\bar{a}^1(7)}(8) = 1) - P_n(Y_{\bar{a}^0(7)}(8) = 1)$.

Inferences for the AUC and RD effect measures were derived from prior work[5] based on the delta method and the influence curve of the IPW estimator β_n.

eMethods 4 - Denominator of the Inverse Probability Weights

The conditional probabilities $P(A(t) = a^k(t) \mid \bar{L}(t), \bar{Y}(t) = 0, \bar{A}(t-1) = \bar{a}^k(t-1))$ for $t = 0, \ldots, 9$ and $k = 0, 1$ that define the denominators of the IP weights used to fit the MSMs described above can be factorized based on the following 10 propensity scores (PS) for:

- baseline initiation of analog-containing insulin therapy denoted by $\mu_1(0)$:

$$P\Big(A_1(0) = 1 \Big| L(0)\Big)$$

- right-censoring due to administrative end of study denoted by $\mu_2(t)$:

$$P\Big(I\Big(A_2(t) = 1, \Gamma = 1\Big) = 1 \Big| \bar{L}(t), \bar{Y}(t) = 0, A_1(0), \bar{A}_2(t-1) = 0\Big)$$

- right-censoring due to disenrollment from the health plan denoted by $\mu_3(t)$:

$$P\Big(I\Big(A_2(t) = 1, \Gamma = 2\Big) = 1 \Big| \bar{L}(t), \bar{Y}(t) = 0, A_1(0), \bar{A}_2(t-1) = 0, I\Big(A_2(t) = 1, \Gamma = 1\Big) = 0\Big)$$

© 2020 Neugebauer R et al. *JAMA Network Open.*

- right-censoring due to start of pregnancy denoted by $\mu_4(t)$:

$$P\left(I\left(A_2(t)=1,\Gamma=3\right)=1\middle|\bar{L}(t),L_{♀}(0)=1,\bar{Y}(t)=0,A_1(0),\bar{A}_2(t-1)=0,I\left(A_2(t)=1,\Gamma\in\{1,2\}\right)=0\right)$$

where $L_{♀}(0)$ denotes the indicator that the patient is female

- right-censoring due to crossover from analog-containing to human-only insulin therapy denoted by $\mu_5(t)$:

$$P\left(I\left(A_2(t)=1,\Gamma=4\right)=1\middle|\bar{L}(t),\bar{Y}(t)=0,A_1(0)=1,\bar{A}_2(t-1)=0,I\left(A_2(t)=1,\Gamma\in\{1,2,3\}\right)=0\right)$$

- right-censoring due to crossover from human-only to analog-containing insulin therapy denoted by $\mu_6(t)$:

$$P\left(I\left(A_2(t)=1,\Gamma=4\right)=1\middle|\bar{L}(t),\bar{Y}(t)=0,A_1(0)=0,\bar{A}_2(t-1)=0,I\left(A_2(t)=1,\Gamma\in\{1,2,3\}\right)=0\right)$$

- right-censoring due to initiation of a non-standard insulins (animal or inhaled) denoted by $\mu_7(t)$:

$$P\left(I\left(A_2(t)=1,\Gamma=5\right)=1\middle|\bar{L}(t),\bar{Y}(t)=0,A_1(0),\bar{A}_2(t-1)=0,I\left(A_2(t)=1,\Gamma\in\{1,\ldots,4\}\right)=0\right)$$

- right-censoring due to early (i.e., at $t=2$) interruption of insulin therapy denoted by $\mu_8(2)$:

$$P\left(I\left(A_2(2)=1,\Gamma=6\right)=1\middle|\bar{L}(2),\bar{Y}(2)=0,A_1(0),\bar{A}_2(1)=0,I\left(A_2(2)=1,\Gamma\in\{1,\ldots,5\}\right)=0\right)$$

- right-censoring due to late (i.e., at $t>2$) interruption of insulin therapy denoted by $\mu_9(t)$:

$$P\left(I\left(A_2(t)=1,\Gamma=6\right)=1\middle|\bar{L}(t),\bar{Y}(t)=0,A_1(0),\bar{A}_2(t-1)=0,I\left(A_2(t)=1,\Gamma\in\{1,\ldots,5\}\right)=0\right)$$

- right-censoring due to death denoted by $\mu_{10}(t)$:

$$P\left(I\left(A_2(t)=1,\Gamma=7\right)=1\middle|\bar{L}(t),\bar{Y}(t)=0,A_1(0),\bar{A}_2(t-1)=0,I\left(A_2(t)=1,\Gamma\in\{1,\ldots,6\}\right)=0\right).$$

We note that the last PS above is not considered to define the IP weights in the analyses that evaluate all-cause mortality because death is then the failure outcome of interest (i.e., there is no right-censoring due to death). For the AMI, CHF, CVA, and CVD mortality outcomes, we constructed the denominators of the IP weights for all outcomes contributing to the MSM fits as follows for $t=0,\ldots,9$: $\mu_1(0)^{A_1(0)}(1-\mu_1(0))^{1-A_1(0)}\prod_{j=0}^{t}(1-\mu_2(j))(1-\mu_3(j))(1-\mu_4(j))^{L_{♀}(0)}(1-\mu_5(j))^{A_1(0)}(1-\mu_6(j))^{1-A_1(0)}(1-\mu_7(j))(1-\mu_8(2))^{I(j=2)}(1-\mu_9(j))^{I(j>2)}(1-\mu_{10}(j))$.

Each of the first three approaches considered for estimating these denominators of the IP weights consists in fitting a separate logistic model for each of the the 10 PS $\mu_j(t)$ just described. The three approaches only differ by the set of covariates that define each of the main terms included in each logistic model. We describe these sets in the next section.

© 2020 Neugebauer R et al. *JAMA Network Open.*

eMethods 5 - Standard Propensity Score Estimation with Three Covariate Adjustment Sets

In the first approach implemented to estimate the denominators of the IP weights, the main terms included in a given PS logistic model were those associated with covariates presumed to impact both failure and the PS outcome as indicated in eTables 4-5. For instance, in the analyses of CHF, the PS logistic model for baseline initiation of analog-containing (versus human-only) insulin therapy included main terms for all covariates in these tables where a value of 1 is found in both the $\mu_1(0)$ and CHF columns. For the time-dependent covariates selected based on this rationale, only main terms for their current values $L(t)$ were included in the PS logistic models, i.e., no main terms for other summary measures of the covariate histories were considered (e.g., latest change in value $L(t) - L(t-1)$ or a lagged value $L(t-1)$). In addition, all PS logistic models except for non-standard insulin initiation included main terms for the patient's age at index date and the PS logistic model for $\mu_1(0)$ also included main terms for and interaction terms between the dummy variables that encode health plan membership (i.e., HP, KPCO, KPNC, or KPSC) and the index date year. All PS logistic models fitted with pooled data over time (i.e., $\mu_j(t)$ for $j = 2, \ldots, 7, 9, 10$) also included main terms for time t (expressed in 90-day intervals). In addition, except for the PS logistic model for $\mu_1(0)$, all other PS models included a main term for the baseline insulin therapy $A_1(0)$. For the PS logistic models for administrative end of study and start of pregnancy, only main terms for age at index, t, and $A_1(0)$ were included in the models. For the PS logistic model for the initiation of non-standard insulins, only main terms for t and $A_1(0)$ were included in the model because <5 patients initiated non-standard insulins which limited the number of covariate that could be considered. All continuous variables considered by the various PS logistic models were discretized using the cutoffs given in eTable 6 and main terms for the resulting dummy variables (for the non-reference level) were included in the models. eTable 7 provides an example of the logistic model fit for $\mu_5(t)$ based on the PS estimation approach 1.

The second approach implemented to estimate the denominators of the IP weights followed the same principles with the difference that the main terms included in a given PS logistic model (including for start of pregnancy and administrative end of study) were those associated with covariates presumed to, at least, impact failure as indicated in eTables 4-5. However, for the PS logistic model for the initiation of non-standard insulins, only main terms for t and $A_1(0)$ were included in the model because <5 patients initiated non-standard insulins which limited the number of covariate that could be considered. All other modeling decisions were identical to those of the first approach described above. eTables 8-9 provide an example of the logistic model fit for $\mu_5(t)$ based on the PS estimation approach 2.

The third approach implemented to estimate the denominators of the IP weights followed the same principles with the difference that the main terms included in a given PS logistic model were those associated with the covariates presumed to impact either failure or the PS outcome as indicated in eTables 4-5. The PS logistic models for the start of pregnancy and administrative end of study included main terms for all covariates presumed to affect failure. However, for the PS logistic model for the initiation of non-standard insulins, only main terms for t and $A_1(0)$ were included in the model because <5 patients initiated non-standard insulins which limited the number of covariate that could be considered. All other modeling decisions were identical to those of the first approach described above. eTables 10-11 provide an example of the logistic model fit for $\mu_5(t)$ based on the PS estimation approach 3.

Thus, the three sets of variables that define the main terms included in any given PS logistic model according to the three approaches just described are nested and of increasing size.

eMethods 6 - Data-adaptive Propensity Score Estimation

In the fourth approach implemented to estimate the denominators of the IP weights, a separate super learner[6] was used to estimate each of the 10 PS $\mu_j(t)$ instead of a separate logistic model (as done in the first three approaches). Each super learner was constructed based on 10-fold cross-validation and three learners corresponding with the same three logistic models considered in the first three PS estimation approaches described above. eTable 12 provides an example of the super learner fit for $\mu_5(t)$ based on the PS estimation approach 4.

eMethods 7 - Results

eTable 13 describes the proportions of patients initiating HI versus AI therapy by site and year of study entry for patients in the main cohort. This table indicates that the great majority of patients from site 4 were first prescribed AI with little fluctuation over the years of the study. This is in contrast to the other 3 sites where most patients were first prescribed HI with relatively little temporal fluctuation at sites 2 and 3, but more temporal fluctuation at site 1 in insulin prescription patterns over the years of the study. Results from eTable 13 motivated the conduct of two sets of sensitivity analyses using, first, only the subset of patients from sites 1-3 (125,257), and second, only the subset of

© 2020 Neugebauer R et al. *JAMA Network Open.*

patients from site 1 (64,092).

The distributions of follow-up times by exposure regimen for each of the five primary analyses are described in eTables 14-28.

Results of all primary and sensitivity analyses implemented with the four PS estimation approaches described above along with their corresponding unadjusted analyses (i.e., same models fitted without weights) are displayed in eTables 29, 30, 31, 32, and 33 for AMI, CHF, CVA, CVD-mortality, and all-cause mortality, respectively. Inference for the hazard ratio is given in the column “HR” and derived from the MSM fit that assumes constant hazard ratios over time (proportionality assumption). Inference in the “AUC”, “RD1”, and “RD2” columns are derived from the same saturated MSM fit. The “AUC” column contains the p-value from the statistical test that the area between the survival curves is equal to 0. The “RD1” and “RD2” columns provide inferences for the cumulative risk differences at 1 and 2 years (i.e., 4 and 8 quarters) after the index date. 95% confidence intervals for the HR and RDs are given in between squared brackets, standard errors are given by “SE”, and the p-values of the statistical tests that HR=1/RD=0 are given by “p”. We note that p-values were not adjusted for multiple testing. The crude (i.e., unadjusted) and SL-based IPW estimates of the counterfactual survival curves associated with the AUC p-values given in the eTables are displayed in eFigures 1-5. Summary statistics for the inverse probability weights involved in all primary and sensitivity analyses are displayed in eTables 34, 35, 36, 37, and 38 for AMI, CHF, CVA, CVD-mortality, and all-cause mortality, respectively.

Null findings from the primary PP analyses are generally supported by the adjusted estimates from sensitivity PP analyses. CHF results from the site 1 sensitivity analyses based on PS estimation with logistic models using covariate sets 2 and 3 and data-adaptive PS estimation with SL provided the greatest statistical evidence of a potential difference between the two exposure regimens considered and suggest a potential beneficial effect of AI against CHF, but not all cause mortality, CVD, MI, or CVA.

© 2020 Neugebauer R et al. *JAMA Network Open.*

eTable 1: Sources of Data and Codes Used to Ascertain Major Cardiovascular Events and Mortality.

**Fatal or Nonfatal Myocardial Infarction** (including Acute Coronary Syndrome)	ICD-9-CM codes : 410.xx	Inpatient hospital discharges (principle discharge diagnosis)
**Fatal or Nonfatal Stroke** Ischemic stroke Hemorrhagic stroke	ICD-9-CM codes : 430.xx, 431.xx, 433.x1, 434.x1	Inpatient hospital discharges (principle discharge diagnosis)
**Hospitalization for Heart Failure (**discharged either alive or deceased)	ICD-9-CM codes: 402.01, 402.11, 402.91, 404.01, 404.03, 404.11, 404.13, 404.91, 404.93, 428.xx	Inpatient hospital discharges (principle discharge diagnosis)
**Cardiovascular Mortality**	ICD9/ICD10 groups: CHD,HF: 50,51,52,53,54,55,58,59,60 CeVD: 61 PAD/Arteriosclerosis: 62,63	Social Security Admin; National Death Index; State Death Records; Tumor Registry data; Encounter data; Patient data; Membership data
**Overall Mortality**	--	Social Security Admin; National Death Index; State Death Records; Tumor Registry data; Encounter data; Patient data; Membership data
CHD: coronary heart disease; HF: heart failure; CeVD: cerebrovascular disease; PAD: peripheral arterial disease.		

© 2020 Neugebauer R et al. *JAMA Network Open.*

eTable 2: Part I of II - Brief description of all attributes (L) in the covariate adjustment sets.

Covariate handle	Brief covariate definition
afib	atrial fibrillation
age.at.index	age at index date
alcoholabuse	alcohol abuse
anemia	anemia
anticoag	anticoagulant medication
anxiety	anxiety
asthma	asthma
bariatric	bariatric surgery
bipolar	bipolar affective disorder
bmi	body mass index (Kg/m2)
cabg	coronary artery bypass graft
cad	coronary artery disease
cancer	cancer other than non-melanoma skin cancer
census.hsgrad	high school graduate
census.medhhincome	median household income
cevd	cerebrovascular disease
chf	congestive heart failure
chf.event	CHF hospitlaization
ckd	chronic kidney disease
composite.protein	urine microalbumin creatinine ratio
connective	vasculitis/connective tissue disease
copd	chronic obstructive lung disease
dementia	dementia
depression	depression
diastolic	diastolic blood pressure
dpp4	DPP-4 class of glucose-lowering medication
drugabuse.alt	substance abuse disorder (other than alcohol)
drugcount	total number of prescription medications
early.adopter	use of DPP-4, GLT-1, SGLTs within 5 years from FDA approval
elixhauser	Elixhauser comorbidity score
flag.incident	incident diabetes (diabetes recognition date $\geq$ 18 months since health plan enrollment)
gender	gender
gfr	glomerular filtration rate cc/min/2.72m2
glp1	GLP-1 agonist glucose lowering medication
hdl	high-denisty lipoprotein cholesterol
hgba1c	hemoglobin A1c value (%)

© 2020 Neugebauer R et al. *JAMA Network Open.*

eTable 3: Part II of II - Brief description of all attributes (L) in the covariate adjustment sets.

Covariate handle	Brief covariate definition
hiv	HIV infection
hmosite	study site
htnmed	hypertension medication
hyperglycemia	hyperglycemia diagnosis code
hyperlipidemia	dyslipidemia
hypertension	hypertension
hypoglycemia	hypoglycemia diagnosis code
index.year	index year
insulin.rxmd.analogpct	% AI dispensings in last year for index insulin prescriber
insulin.rxmd.spec	prescribing provider specialty for insulin dispensed at index
insulin.rxmd.type	index insulin provider type (NP/PA versus MD/PO)
insulin.rxmd.yrs	years since prescribing provider of index insulin graduated
ip.count	number of inpatient encounters
ldl	low-denisty lipoprotein cholesterol
lipidmed	cholesterol medication
mavalvedisorder	mitral or aortic valve heart disease
met	metformin glucose lowering medication
mi.event	myocardial infarction
neurodisorder	neuromuscular disorder
nitrate	nitrate medication
platemed	platelet inhibitor medication
ptvalvedisorder	pulmonic or tricuspid valve heart disease
pvd	peripheral vascular disease
racegrp	race group
retinopathy	retinopathy
schizophrenia	schizophrenia
sglt2	SGLT2 inhibitor class of glucose lowering medication
smoking.status	smoking status
stent	stent placed in coronary artery
stroke.event	stroke event
sul	sulfonylurea glucose lowering medication
systolic	systolic blood pressure
tzd	TZD glucose lowering medication
years.since.dm	duration of diabetes in years

© 2020 Neugebauer R et al. *JAMA Network Open.*

eTable 4: Part I of II - List of covariates considered in the various analyses and whether they are assumed to impact exposure decisions, censoring events, or outcomes.

Covariate	AMI	CHF	CVA	CVD	Death $\mu_{10}(t)$	Initial insulin $\mu_1(0)$	Insurance coverage $\mu_3(t)$	Adherence to initial insulin $\mu_j(t)$ for $j = 5, 6, 8, 9$	Time-dependent
afib	1	1	1	1	1	0	0	0	1
age.at.index	1	1	1	1	1	1	1	1	0
alcoholabuse	1	1	1	1	1	0	1	1	1
anemia	1	1	1	1	1	0	0	0	1
anticoag	1	1	1	1	1	0	0	0	1
anxiety	1	1	1	1	1	0	0	1	1
asthma	1	1	1	1	1	0	0	0	1
bariatric	1	0	1	0	0	0	1	0	1
bipolar	1	1	1	1	1	0	1	1	1
bmi	1	1	1	1	1	0	0	0	1
cabg	1	1	1	1	1	0	1	1	1
cad	1	1	1	1	1	0	1	1	1
cancer	0	0	0	0	0	0	1	0	1
census.hsgrad	1	1	1	1	1	0	1	1	0
census.medhhincome	1	1	1	1	1	0	1	1	0
cevd	1	1	1	1	1	0	1	1	1
chf	1	1	1	1	1	0	1	1	1
chf.event	1	1	1	1	1	0	1	1	1
ckd	1	1	1	1	1	0	1	0	1
composite.protein	1	1	1	1	1	0	0	0	1
connective	1	1	1	1	1	0	1	0	1
copd	1	1	1	1	1	0	1	0	1
dementia	1	1	1	1	1	1	1	1	1
depression	1	1	1	1	1	0	1	1	1
diastolic	1	1	1	0	0	0	0	0	1
dpp4	1	1	1	1	1	1	0	0	1
drugabuse.alt	1	1	1	1	1	0	1	1	1
drugcount	0	0	0	0	0	0	0	1	0
early.adopter	0	0	0	0	0	1	0	0	0
elixhauser	0	0	0	0	0	0	1	1	0
flag.incident	1	1	1	1	1	0	0	0	0
gender	1	1	1	1	1	0	0	1	0
gfr	1	1	1	1	1	0	0	0	1
glp1	1	1	1	1	1	0	0	0	1
hdl	1	0	1	1	0	0	0	0	1
hgba1c	1	1	1	1	1	1	0	1	1

© 2020 Neugebauer R et al. *JAMA Network Open.*

eTable 5: Part II of II - List of covariates considered in the various analyses and whether they are assumed to impact exposure decisions, censoring events, or outcomes.

Covariate	AMI	CHF	CVA	CVD	Death $\mu_{10}(t)$	Initial insulin $\mu_1(0)$	Insurance coverage $\mu_3(t)$	Adherence to initial insulin $\mu_j(t)$ for $j = 5, 6, 8, 9$	Time-dependent
hiv	1	1	1	1	1	0	1	0	1
hmosite	0	0	0	0	0	1	1	0	0
htnmed	1	1	1	1	0	0	0	0	1
hyperglycemia	1	1	1	1	1	1	0	1	1
hyperlipidemia	1	1	1	1	1	0	0	0	1
hypertension	1	1	1	1	1	0	1	0	1
hypoglycemia	1	1	1	1	1	1	0	1	1
index.year	0	0	0	0	0	1	0	0	0
insulin.rxmd.analogpct	0	0	0	0	0	1	0	0	0
insulin.rxmd.spec	0	0	0	0	0	1	0	0	0
insulin.rxmd.type	0	0	0	0	0	1	0	0	0
insulin.rxmd.yrs	0	0	0	0	0	1	0	0	0
ip.count	1	1	1	1	1	0	1	0	1
ldl	1	0	1	1	0	0	0	0	1
lipidmed	1	0	1	1	0	0	0	0	1
mavalvedisorder	0	1	1	1	1	0	0	0	1
met	1	1	1	1	1	0	0	0	1
mi.event	1	1	1	1	1	0	1	0	1
neurodisorder	0	0	0	0	0	0	1	0	1
nitrate	1	1	1	1	1	0	0	0	1
platemed	1	1	1	1	1	0	0	0	1
ptvalvedisorder	0	1	0	1	1	0	0	0	1
pvd	1	1	1	1	1	0	1	0	1
racegrp	1	1	1	1	1	0	1	1	0
retinopathy	1	1	1	1	1	0	0	0	1
schizophrenia	1	1	1	1	1	0	1	1	1
sglt2	1	1	1	1	1	1	0	0	1
smoking.status	1	1	1	1	1	0	0	1	0
stent	1	1	1	1	1	0	1	1	1
stroke.event	1	1	1	1	1	0	1	1	1
sul	1	1	1	1	1	1	0	0	1
systolic	1	1	1	1	0	0	0	0	1
tzd	1	1	1	1	1	1	0	0	1
years.since.dm	1	1	1	1	1	0	0	0	0

© 2020 Neugebauer R et al. *JAMA Network Open.*

eTable 6: Cutoffs used to discretize continuous covariates.

Variable	Cutoffs
age.at.index (years)	35;45;55;65;75
bmi (Kg/m^2)	18.5; 25; 30; 35; 40
census.hsgrad	0.5
census.medhhincome	30000;50000;70000;90000
diastolic (mm Hg)	80;90;100
drugcount	2;3;4;5;6;7;8;9
elixhauser score	1;3;5
gfr (mL/1.73 m^2/min)	15; 30; 45; 60; 90
hdl (mg/dL)	40;50;60
hgba1c (%)	7; 7.5; 8; 8.5;9;10
insulin.rxmd.analogpct	0.1;0.5;0.9
insulin.rxmd.yrs	5;20
ip.count	1;2
ldl (mg/dL)	70;100;130
systolic (mm Hg)	120;140;160
t	1;2;3;4;6;8;10;12;16
years.since.dm	1;6;10

© 2020 Neugebauer R et al. *JAMA Network Open.*

eTable 7: PS estimation approach 1 in AMI primary analysis: Logistic model for the probability of right-censoring due to a switch from analog-containing to human-only insulin therapy. Model fitted with 126617 observations from 18318 unique patients. Reference categories: t$<$1, 55$\leq$age.at.index$<$65, genderM, racegrp6:WHITE, smoking.statusNEVER/UNK, hgba1c$<$7, 50000$\leq$census.medhhincome$<$70000, census.hsgrad$\geq$0.5. Indicators of missing covariate measurement are denoted by I.* (e.g., I.hgba1c denotes the absence of hgba1c monitoring at quarter 't').

Covariate	Coef	OR
(Intercept)	-3.781	
t in [1;2[	-0.156	0.855
t in [2;3[	0.377	1.457
t in [3;4[	0.039	1.039
t in [4;6[	2e-03	1.002
t in [6;8[	4e-03	1.004
t in [8;10[	0.107	1.113
t in [10;12[	0.089	1.093
t in [12;16[	0.265	1.304
t $\geq$16	0.303	1.354
age.at.index <35	-0.101	0.904
age.at.index in [35;45[	1e-03	1.001
age.at.index in [45;55[	-0.036	0.964
age.at.index in [65;75[	0.094	1.098
age.at.index $\geq$75	0.141	1.152
genderF	0.096	1.101
racegrp1:HISPANIC	0.188	1.206
racegrp2:BLACK	-0.016	0.984
racegrp3:HI/PI	0.38	1.462
racegrp4:ASIAN	0.127	1.136
racegrp5:NATIV	-0.115	0.892
racegrp7:MISS	-0.044	0.957
smoking.statusCURRENT	-6e-02	0.942
smoking.statusPAST	0.049	1.051
cabg	-0.089	0.914
cad	2e-03	1.002
cevd	0.106	1.112

Covariate	Coef	OR
chf	0.068	1.07
stent	-0.135	0.874
chf.event	0.303	1.354
hgba1c in [7;7.5[	-0.103	0.902
hgba1c in [7.5;8[	0.318	1.374
hgba1c in [8;8.5[	0.696	2.005
hgba1c in [8.5;9[	0.754	2.126
hgba1c in [9;10[	0.955	2.599
hgba1c $\geq$10	1.006	2.735
stroke.event	0.429	1.535
I.hgba1c	-1.414	0.243
alcoholabuse	0.098	1.103
bipolar	0.113	1.12
depression	0.087	1.09
drugabuse.alt	0.315	1.37
schizophrenia	0.139	1.149
census.medhhincome <30000	0.114	1.12
census.medhhincome in [30000;50000[	0.068	1.07
census.medhhincome in [70000;90000[	4e-02	1.041
census.medhhincome $\geq$90000	0.039	1.039
census.hsgrad <0.5	0.057	1.059
anxiety	0.033	1.034
dementia	0.193	1.213
hyperglycemia	0.272	1.313
hypoglycemia	0.779	2.179
I.census.medhhincome	-10.214	0
I.census.hsgrad	9.496	13300.155

© 2020 Neugebauer R et al. *JAMA Network Open.*

eTable 8: PS estimation approach 2 in AMI primary analysis (Part I of II): Logistic model for the probability of right-censoring due to a switch from analog-containing to human-only insulin therapy. Model fitted with 126617 observations from 18318 unique patients. Reference categories: 55≤age.at.index<65, ip.count<1, 60≤gfr<90, hdl<40, hgba1c<7, 70≤ldl<100, 120≤systolic<140, 6≤years.since.dm<10, diastolic<80, 50000≤census.medhhincome<70000, census.hsgrad≥0.5, 30≤bmi<35, t<1, genderM, racegrp6:WHITE, smoking.statusNEVER/UNK, flag.incidentUnknown, composite.protein1:NORML. Indicators of missing covariate measurement are denoted by I.* (e.g., I.hgba1c denotes the absence of hgba1c monitoring at quarter 't').

Covariate	Coef	OR
(Intercept)	-4.371	
age.at.index <35	0.099	1.104
age.at.index in [35;45[	0.094	1.099
age.at.index in [45;55[	0.017	1.017
age.at.index in [65;75[	2e-03	1.002
age.at.index ≥75	3e-03	1.003
genderF	0.074	1.077
racegrp1:HISPANIC	0.159	1.172
racegrp2:BLACK	-0.033	0.968
racegrp3:HI/PI	0.342	1.408
racegrp4:ASIAN	0.1	1.105
racegrp5:NATIV	-0.082	0.922
racegrp7:MISS	0.092	1.097
smoking.statusCURRENT	-0.087	0.917
smoking.statusPAST	0.018	1.019
mi.event	0.17	1.185
bariatric	-1.079	0.34
cabg	-0.262	0.769
cad	-0.108	0.897
cevd	-0.061	0.941
chf	-0.104	0.901
hyperlipidemia	0.102	1.107
hypertension	0.071	1.073
pvd	-0.15	0.861
stent	-0.29	0.748
chf.event	0.112	1.118
htnmed	0.191	1.211
ip.count in [1;2[	0.24	1.272
ip.count ≥2	0.458	1.581

Covariate	Coef	OR
lipidmed	0.125	1.133
nitrate	0.062	1.064
platemed	-0.021	0.979
gfr <15	-0.281	0.755
gfr in [15;30[	0.025	1.025
gfr in [30;45[	9e-02	1.094
gfr in [45;60[	5e-02	1.051
gfr ≥90	0.036	1.037
hdl in [40;50[	-0.061	0.941
hdl in [50;60[	-0.032	0.969
hdl ≥60	-0.053	0.948
hgba1c in [7;7.5[	-4e-02	0.961
hgba1c in [7.5;8[	0.402	1.494
hgba1c in [8;8.5[	0.789	2.2
hgba1c in [8.5;9[	0.853	2.348
hgba1c in [9;10[	1.057	2.878
hgba1c ≥10	1.14	3.127
ldl <70	-0.022	0.978
ldl in [100;130[	-0.071	0.931
ldl ≥130	-0.042	0.959
stroke.event	0.418	1.519
systolic <120	-0.062	0.939
systolic in [140;160[	-0.124	0.884
systolic ≥160	-0.112	0.894
I.gfr	-0.121	0.886
I.hdl	-0.179	0.836
I.hgba1c	-0.814	0.443
I.ldl	-0.018	0.982
I.systolic	-0.4	0.67

© 2020 Neugebauer R et al. *JAMA Network Open.*

eTable 9: PS estimation approach 2 in AMI primary analysis (Part II of II): Logistic model for the probability of right-censoring due to a switch from analog-containing to human-only insulin therapy. Model fitted with 126617 observations from 18318 unique patients. Reference categories: 55≤age.at.index<65, ip.count<1, 60≤gfr<90, hdl<40, hgba1c<7, 70≤ldl<100, 120≤systolic<140, 6≤years.since.dm<10, diastolic<80, 50000≤census.medhhincome<70000, census.hsgrad≥0.5, 30≤bmi<35, t<1, genderM, racegrp6:WHITE, smoking.statusNEVER/UNK, flag.incidentUnknown, composite.protein1:NORML. Indicators of missing covariate measurement are denoted by I.* (e.g., I.hgba1c denotes the absence of hgba1c monitoring at quarter 't').

Covariate	Coef	OR
years.since.dm <1	0.048	1.049
years.since.dm in [1;6[	-0.106	0.9
years.since.dm ≥10	0.188	1.207
flag.incidentNo	0.263	1.3
flag.incidentYes	0.193	1.213
alcoholabuse	-5e-03	0.995
bipolar	0.059	1.061
connective	-0.047	0.954
depression	1e-03	1.001
drugabuse.alt	0.219	1.245
hiv	-0.314	0.73
retinopathy	8e-03	1.008
schizophrenia	0.059	1.061
diastolic in [80;90[	-0.018	0.982
diastolic in [90;100[	-0.159	0.853
diastolic ≥100	-0.177	0.837
I.diastolic	0.028	1.029
census.medhhincome <30000	0.111	1.118
census.medhhincome in [30000;50000[	0.065	1.067
census.medhhincome in [70000;90000[	0.043	1.044
census.medhhincome ≥90000	0.045	1.046
census.hsgrad <0.5	0.057	1.058
afib	-0.043	0.958
anxiety	-0.045	0.956
asthma	0.047	1.048
ckd	0.175	1.191
copd	0.254	1.289
dementia	0.194	1.214
anemia	0.184	1.202

Covariate	Coef	OR
dpp4	-0.524	0.592
glp1	-0.565	0.568
met	3e-02	1.031
anticoag	0.095	1.099
sglt2	-9.926	0
sul	0.146	1.157
tzd	-0.111	0.895
bmi <18.5	0.208	1.232
bmi in [18.5;25[	-0.072	0.93
bmi in [25;30[	-0.023	0.977
bmi in [35;40[	-4e-03	0.996
bmi ≥40	0.038	1.039
composite.protein0:UNK	-0.131	0.877
composite.protein2:MICRO	6e-03	1.006
composite.protein3:MACRO	-0.104	0.901
hyperglycemia	0.172	1.188
hypoglycemia	0.665	1.944
I.census.medhhincome	-10.209	0
I.census.hsgrad	9.671	15855.835
I.bmi	-0.379	0.685
I.composite.protein	-0.331	0.718
t in [1;2[	0.045	1.046
t in [2;3[	0.662	1.939
t in [3;4[	0.312	1.366
t in [4;6[	0.25	1.283
t in [6;8[	0.242	1.273
t in [8;10[	0.334	1.397
t in [10;12[	0.325	1.384
t in [12;16[	0.51	1.665
t ≥16	0.556	1.744

© 2020 Neugebauer R et al. *JAMA Network Open.*

eTable 10: PS estimation approach 3 in AMI primary analysis (Part I of II): Logistic model for the probability of right-censoring due to a switch from analog-containing to human-only insulin therapy. Model fitted with 126617 observations from 18318 unique patients. Reference categories: 55$\leq$age.at.index$<$65, genderM, racegrp6:WHITE, 50000$\leq$census.medhhincome$<$70000, census.hsgrad$\geq$0.5, smoking.statusNEVER/UNK, elixhauser$\geq$5, drugcountgeq9, hgba1c$<$7, ip.count$<$1, 60$\leq$gfr$<$90, hdl$<$40, 70$\leq$ldl$<$100, 120$\leq$systolic$<$140, 6$\leq$years.since.dm$<$10, flag.incidentUnknown, diastolic$<$80, 30$\leq$bmi$<$35, composite.protein1:NORML, t$<$1. Indicators of missing covariate measurement are denoted by I.* (e.g., I.hgba1c denotes the absence of hgba1c monitoring at quarter ’t’).

Covariate	Coef	OR
(Intercept)	-4.508	
age.at.index <35	0.085	1.089
age.at.index in [35;45[	0.091	1.095
age.at.index in [45;55[	0.017	1.017
age.at.index in [65;75[	7e-03	1.007
age.at.index $\geq$75	7e-03	1.007
genderF	0.079	1.082
racegrp1:HISPANIC	0.155	1.168
racegrp2:BLACK	-0.032	0.969
racegrp3:HI/PI	0.341	1.406
racegrp4:ASIAN	0.101	1.106
racegrp5:NATIV	-0.078	0.925
racegrp7:MISS	9e-02	1.094
census.medhhincome <30000	0.11	1.116
census.medhhincome in [30000;50000[	0.062	1.064
census.medhhincome in [70000;90000[	0.043	1.043
census.medhhincome $\geq$90000	0.047	1.048
census.hsgrad <0.5	5e-02	1.052
smoking.statusCURRENT	-0.088	0.915
smoking.statusPAST	0.021	1.021
elixhauser in [1;3[	-0.053	0.948
elixhauser in [3;5[	2e-02	1.02
drugcount <2	0.212	1.237
drugcount in [2;3[	0.17	1.185
drugcount in [3;4[	0.214	1.239
drugcount in [4;5[	0.071	1.074
drugcount in [5;6[	0.062	1.064
drugcount in [6;7[	0.051	1.052
drugcount in [7;8[	0.036	1.036
drugcount in [8;9[	0.066	1.069
alcoholabuse	-5e-03	0.995
anxiety	-0.038	0.963

Covariate	Coef	OR
bipolar	0.066	1.068
cabg	-0.257	0.773
cad	-0.103	0.902
cevd	-6e-02	0.942
chf	-0.093	0.911
dementia	0.193	1.213
depression	7e-03	1.007
drugabuse.alt	0.226	1.254
schizophrenia	0.067	1.07
stent	-0.292	0.747
chf.event	0.11	1.117
hgba1c in [7;7.5[	-0.043	0.958
hgba1c in [7.5;8[	0.396	1.486
hgba1c in [8;8.5[	0.783	2.188
hgba1c in [8.5;9[	0.849	2.337
hgba1c in [9;10[	1.05	2.858
hgba1c $\geq$10	1.131	3.1
hyperglycemia	0.167	1.182
hypoglycemia	0.664	1.943
stroke.event	0.424	1.529
I.census.hsgrad	9.67	15833.047
I.census.medhhincome	-10.21	0
I.hgba1c	-0.812	0.444
mi.event	0.175	1.191
bariatric	-1.075	0.341
hyperlipidemia	0.116	1.123
hypertension	0.071	1.073
pvd	-0.15	0.86
htnmed	0.21	1.234
ip.count in [1;2[	0.246	1.279
ip.count $\geq$2	0.468	1.598
lipidmed	0.136	1.146

© 2020 Neugebauer R et al. *JAMA Network Open.*

eTable 11: PS estimation approach 3 in AMI primary analysis (Part II of II): Logistic model for the probability of right-censoring due to a switch from analog-containing to human-only insulin therapy. Model fitted with 126617 observations from 18318 unique patients. Reference categories: 55$\leq$age.at.index$<$65, genderM, racegrp6:WHITE, 50000$\leq$census.medhhincome$<$70000, census.hsgrad$\geq$0.5, smoking.statusNEVER/UNK, elixhauser$\geq$5, drugcountgeq9, hgba1c$<$7, ip.count$<$1, 60$\leq$gfr$<$90, hdl$<$40, 70$\leq$ldl$<$100, 120$\leq$systolic$<$140, 6$\leq$years.since.dm$<$10, flag.incidentUnknown, diastolic$<$80, 30$\leq$bmi$<$35, composite.protein1:NORML, t$<$1. Indicators of missing covariate measurement are denoted by I.* (e.g., I.hgba1c denotes the absence of hgba1c monitoring at quarter ’t’).

Covariate	Coef	OR
nitrate	0.071	1.074
platemed	-0.014	0.986
gfr <15	-0.28	0.756
gfr in [15;30[	0.037	1.037
gfr in [30;45[	0.1	1.105
gfr in [45;60[	0.055	1.057
gfr $\geq$90	0.031	1.031
hdl in [40;50[	-0.062	0.94
hdl in [50;60[	-0.033	0.967
hdl $\geq$60	-0.055	0.946
ldl <70	-2e-02	0.981
ldl in [100;130[	-0.074	0.928
ldl $\geq$130	-0.049	0.953
systolic <120	-0.062	0.94
systolic in [140;160[	-0.122	0.885
systolic $\geq$160	-0.108	0.898
I.gfr	-0.122	0.885
I.hdl	-0.178	0.837
I.ldl	-0.019	0.981
I.systolic	-0.379	0.685
years.since.dm <1	0.044	1.045
years.since.dm in [1;6[	-0.107	0.899
years.since.dm $\geq$10	0.188	1.207
flag.incidentNo	0.261	1.298
flag.incidentYes	0.196	1.217
connective	-4e-02	0.961
hiv	-0.318	0.727
retinopathy	5e-03	1.006
diastolic in [80;90[	-0.019	0.981
diastolic in [90;100[	-0.16	0.852
diastolic $\geq$100	-0.179	0.836
I.diastolic	9e-03	1.009

Covariate	Coef	OR
afib	-0.035	0.966
asthma	0.054	1.055
ckd	0.174	1.19
copd	0.265	1.303
anemia	0.19	1.21
dpp4	-0.508	0.602
glp1	-0.557	0.573
met	0.049	1.05
anticoag	0.101	1.107
sglt2	-9.909	0
sul	0.168	1.182
tzd	-0.093	0.911
bmi <18.5	0.205	1.227
bmi in [18.5;25[	-7e-02	0.932
bmi in [25;30[	-0.022	0.979
bmi in [35;40[	-1e-03	0.999
bmi $\geq$40	4e-02	1.041
composite.protein0:UNK	-0.122	0.885
composite.protein2:MICRO	7e-03	1.007
composite.protein3:MACRO	-0.101	0.904
I.bmi	-0.378	0.685
I.composite.protein	-0.331	0.718
t in [1;2[	0.037	1.038
t in [2;3[	0.656	1.927
t in [3;4[	0.307	1.36
t in [4;6[	0.245	1.277
t in [6;8[	0.237	1.268
t in [8;10[	0.329	1.39
t in [10;12[	0.32	1.378
t in [12;16[	0.505	1.657
t $\geq$16	0.552	1.736

© 2020 Neugebauer R et al. *JAMA Network Open.*

eTable 12: PS estimation approach 4 in AMI primary analysis: Super learner estimator for the probability of right-censoring due to a switch from analog-containing to human-only insulin therapy. Estimator derived based on 126617 observations from 18318 unique patients. Three learners were considered corresponding with three logistic models described in eTables 7-11. The weighted average (SL weights) of the 3 learners that define the super learner was constructed based on 10-fold cross-validation (CV).

	Logistic model 1 (eTable 7)	Logistic model 2 (eTables 8-9)	Logistic model 3 (eTables 10-11)
CV risk	0.03492	0.0346	0.0346
SL weights	0.05641	0.61874	0.32485

© 2020 Neugebauer R et al. *JAMA Network Open.*

eTable 13: Distribution of type of initial insulin therapy for patients in the main cohort by site and year of cohort entry.

	Site 1		Site 2		Site 3		Site 4	
**Year**	**Analog %**	**Human %**	**Analog %**	**Human %**	**Analog %**	**Human %**	**Analog %**	**Human %**
2005	11.01	88.99	9.2	90.8	1.26	98.74	88.37	11.63
2006	20.55	79.45	11.94	88.06	2.67	97.33	94.07	5.93
2007	29.57	70.43	9.99	90.01	4.16	95.84	92.48	7.52
2008	34.71	65.29	11.49	88.51	7.13	92.87	95.08	4.92
2009	29.3	70.7	11.51	88.49	5.29	94.71	97.36	2.64
2010	14.76	85.24	7.86	92.14	2.59	97.41	96.55	3.45
2011	8.92	91.08	7.95	92.05	3.92	96.08	98.34	1.66
2012	5.29	94.71	8.52	91.48	3.35	96.65	97.77	2.23
2013	3.99	96.01	8.9	91.1	3.68	96.32	97.92	2.08

© 2020 Neugebauer R et al. *JAMA Network Open.*

eTable 14: Distribution of follow-up time (expressed in 90-day intervals) for patients continuously exposed to analog-containing insulin therapy in the primary AMI analyses (all sites combined).

Follow-up time	Frequency	%	Cumulative Frequency	Cumulative %
[1, 2[	1955	10.33	1955	10.33
[2, 3[	1211	6.40	3166	16.73
[3, 4[	5848	30.90	9014	47.62
[4, 5[	1675	8.85	10689	56.47
[5, 6[	1004	5.30	11693	61.78
[6, 7[	857	4.53	12550	66.30
[7, 8[	761	4.02	13311	70.32
[8, 9[	608	3.21	13919	73.54
[9, 10[	518	2.74	14437	76.27
[10, 11[	464	2.45	14901	78.72
[11, 12[	392	2.07	15293	80.80
[12, 13[	370	1.95	15663	82.75
[13, 17[	1143	6.04	16806	88.79
[17, 21[	879	4.64	17685	93.43
[21, 25[	621	3.28	18306	96.71
[25, 29[	350	1.85	18656	98.56
[29, 33[	192	1.01	18848	99.58
[33, 37[	71	0.38	18919	99.95
$\geq$ 37	9	0.05	18928	100.00
Missing	0	0.00	18928	100.00

© 2020 Neugebauer R et al. *JAMA Network Open.*

eTable 15: Distribution of follow-up time (expressed in 90-day intervals) for patients continuously exposed to human-only insulin therapy in the primary AMI analyses (all sites combined).

Follow-up time	Frequency	%	Cumulative Frequency	Cumulative %
[1, 2[	8951	8.24	8951	8.24
[2, 3[	7098	6.53	16049	14.77
[3, 4[	34325	31.59	50374	46.35
[4, 5[	10653	9.80	61027	56.16
[5, 6[	6156	5.66	67183	61.82
[6, 7[	5322	4.90	72505	66.72
[7, 8[	4523	4.16	77028	70.88
[8, 9[	3848	3.54	80876	74.42
[9, 10[	3044	2.80	83920	77.22
[10, 11[	2711	2.49	86631	79.72
[11, 12[	2412	2.22	89043	81.94
[12, 13[	2161	1.99	91204	83.93
[13, 17[	5977	5.50	97181	89.43
[17, 21[	4112	3.78	101293	93.21
[21, 25[	2888	2.66	104181	95.87
[25, 29[	2354	2.17	106535	98.03
[29, 33[	1270	1.17	107805	99.20
[33, 37[	768	0.71	108573	99.91
≥ 37	99	0.09	108672	100.00
Missing	0	0.00	108672	100.00

eTable 16: Summary statistics of the distribution of follow-up time (expressed in 90-day intervals) by exposure regimen in the primary AMI analyses (all sites combined).

Exposure	N	Min.	1st Qu.	Median	Mean	3rd Qu.	Max.
Analog	18928	1	3	4	6.95	9	37
Human	108672	1	3	4	6.98	9	37

© 2020 Neugebauer R et al. *JAMA Network Open.*

eTable 17: Distribution of follow-up time (expressed in 90-day intervals) for patients continuously exposed to analog-containing insulin therapy in the primary CHF analyses (all sites combined).

Follow-up time	Frequency	%	Cumulative Frequency	Cumulative %
[1, 2[	2023	10.69	2023	10.69
[2, 3[	1243	6.57	3266	17.25
[3, 4[	5808	30.68	9074	47.94
[4, 5[	1667	8.81	10741	56.75
[5, 6[	1003	5.30	11744	62.05
[6, 7[	859	4.54	12603	66.58
[7, 8[	760	4.02	13363	70.60
[8, 9[	605	3.20	13968	73.80
[9, 10[	524	2.77	14492	76.56
[10, 11[	447	2.36	14939	78.93
[11, 12[	392	2.07	15331	81.00
[12, 13[	369	1.95	15700	82.95
[13, 17[	1135	6.00	16835	88.94
[17, 21[	873	4.61	17708	93.55
[21, 25[	605	3.20	18313	96.75
[25, 29[	344	1.82	18657	98.57
[29, 33[	193	1.02	18850	99.59
[33, 37[	69	0.36	18919	99.95
$\geq$ 37	9	0.05	18928	100.00
Missing	0	0.00	18928	100.00

© 2020 Neugebauer R et al. *JAMA Network Open.*

eTable 18: Distribution of follow-up time (expressed in 90-day intervals) for patients continuously exposed to human-only insulin therapy in the primary CHF analyses (all sites combined).

Follow-up time	Frequency	%	Cumulative Frequency	Cumulative %
[1, 2[	9326	8.58	9326	8.58
[2, 3[	7219	6.64	16545	15.22
[3, 4[	34131	31.41	50676	46.63
[4, 5[	10647	9.80	61323	56.43
[5, 6[	6144	5.65	67467	62.08
[6, 7[	5317	4.89	72784	66.98
[7, 8[	4504	4.14	77288	71.12
[8, 9[	3846	3.54	81134	74.66
[9, 10[	3042	2.80	84176	77.46
[10, 11[	2709	2.49	86885	79.95
[11, 12[	2368	2.18	89253	82.13
[12, 13[	2137	1.97	91390	84.10
[13, 17[	5883	5.41	97273	89.51
[17, 21[	4081	3.76	101354	93.27
[21, 25[	2844	2.62	104198	95.88
[25, 29[	2340	2.15	106538	98.04
[29, 33[	1267	1.17	107805	99.20
[33, 37[	768	0.71	108573	99.91
$\geq$ 37	99	0.09	108672	100.00
Missing	0	0.00	108672	100.00

eTable 19: Summary statistics of the distribution of follow-up time (expressed in 90-day intervals) by exposure regimen in the primary CHF analyses (all sites combined).

Exposure	N	Min.	1st Qu.	Median	Mean	3rd Qu.	Max.
Analog	18928	1	3	4	6.90	9	37
Human	108672	1	3	4	6.94	9	37

© 2020 Neugebauer R et al. *JAMA Network Open.*

eTable 20: Distribution of follow-up time (expressed in 90-day intervals) for patients continuously exposed to analog-containing insulin therapy in the primary CVA analyses (all sites combined).

Follow-up time	Frequency	%	Cumulative Frequency	Cumulative %
[1, 2[	1937	10.23	1937	10.23
[2, 3[	1223	6.46	3160	16.69
[3, 4[	5842	30.86	9002	47.56
[4, 5[	1682	8.89	10684	56.45
[5, 6[	1004	5.30	11688	61.75
[6, 7[	853	4.51	12541	66.26
[7, 8[	763	4.03	13304	70.29
[8, 9[	602	3.18	13906	73.47
[9, 10[	514	2.72	14420	76.18
[10, 11[	460	2.43	14880	78.61
[11, 12[	399	2.11	15279	80.72
[12, 13[	368	1.94	15647	82.67
[13, 17[	1136	6.00	16783	88.67
[17, 21[	899	4.75	17682	93.42
[21, 25[	617	3.26	18299	96.68
[25, 29[	359	1.90	18658	98.57
[29, 33[	190	1.00	18848	99.58
[33, 37[	71	0.38	18919	99.95
$\geq$ 37	9	0.05	18928	100.00
Missing	0	0.00	18928	100.00

© 2020 Neugebauer R et al. *JAMA Network Open.*

eTable 21: Distribution of follow-up time (expressed in 90-day intervals) for patients continuously exposed to human-only insulin therapy in the primary CVA analyses (all sites combined).

Follow-up time	Frequency	%	Cumulative Frequency	Cumulative %
[1, 2[	8942	8.23	8942	8.23
[2, 3[	7034	6.47	15976	14.70
[3, 4[	34300	31.56	50276	46.26
[4, 5[	10621	9.77	60897	56.04
[5, 6[	6133	5.64	67030	61.68
[6, 7[	5307	4.88	72337	66.56
[7, 8[	4506	4.15	76843	70.71
[8, 9[	3877	3.57	80720	74.28
[9, 10[	3055	2.81	83775	77.09
[10, 11[	2741	2.52	86516	79.61
[11, 12[	2402	2.21	88918	81.82
[12, 13[	2166	1.99	91084	83.82
[13, 17[	5975	5.50	97059	89.31
[17, 21[	4122	3.79	101181	93.11
[21, 25[	2917	2.68	104098	95.79
[25, 29[	2384	2.19	106482	97.98
[29, 33[	1289	1.19	107771	99.17
[33, 37[	799	0.74	108570	99.91
$\geq$ 37	102	0.09	108672	100.00
Missing	0	0.00	108672	100.00

eTable 22: Summary statistics of the distribution of follow-up time (expressed in 90-day intervals) by exposure regimen in the primary CVA analyses (all sites combined).

Exposure	N	Min.	1st Qu.	Median	Mean	3rd Qu.	Max.
Analog	18928	1	3	4	6.96	9	37
Human	108672	1	3	4	7.01	9	37

© 2020 Neugebauer R et al. *JAMA Network Open.*

eTable 23: Distribution of follow-up time (expressed in 90-day intervals) for patients continuously exposed to analog-containing insulin therapy in the primary CVD mortality analyses (all sites combined).

Follow-up time	Frequency	%	Cumulative Frequency	Cumulative %
[1, 2[	1800	10.95	1800	10.95
[2, 3[	1129	6.87	2929	17.81
[3, 4[	4920	29.92	7849	47.73
[4, 5[	1465	8.91	9314	56.64
[5, 6[	875	5.32	10189	61.97
[6, 7[	792	4.82	10981	66.78
[7, 8[	671	4.08	11652	70.86
[8, 9[	572	3.48	12224	74.34
[9, 10[	501	3.05	12725	77.39
[10, 11[	457	2.78	13182	80.17
[11, 12[	440	2.68	13622	82.84
[12, 13[	414	2.52	14036	85.36
[13, 17[	1231	7.49	15267	92.85
[17, 21[	697	4.24	15964	97.09
[21, 25[	331	2.01	16295	99.10
[25, 29[	137	0.83	16432	99.93
$\geq$ 29	11	0.07	16443	100.00
Missing	0	0.00	16443	100.00

© 2020 Neugebauer R et al. *JAMA Network Open.*

eTable 24: Distribution of follow-up time (expressed in 90-day intervals) for patients continuously exposed to human-only insulin therapy in the primary CVD mortality analyses (all sites combined).

Follow-up time	Frequency	%	Cumulative Frequency	Cumulative %
[1, 2[	7781	9.87	7781	9.87
[2, 3[	6146	7.79	13927	17.66
[3, 4[	25048	31.76	38975	49.42
[4, 5[	7629	9.67	46604	59.10
[5, 6[	4313	5.47	50917	64.57
[6, 7[	3757	4.76	54674	69.33
[7, 8[	3105	3.94	57779	73.27
[8, 9[	2688	3.41	60467	76.68
[9, 10[	2109	2.67	62576	79.35
[10, 11[	1789	2.27	64365	81.62
[11, 12[	1707	2.16	66072	83.79
[12, 13[	1556	1.97	67628	85.76
[13, 17[	4578	5.81	72206	91.57
[17, 21[	3545	4.50	75751	96.06
[21, 25[	1831	2.32	77582	98.38
[25, 29[	1158	1.47	78740	99.85
≥ 29	117	0.15	78857	100.00
Missing	0	0.00	78857	100.00

eTable 25: Summary statistics of the distribution of follow-up time (expressed in 90-day intervals) by exposure regimen in the primary CVD mortality analyses (all sites combined).

Exposure	N	Min.	1st Qu.	Median	Mean	3rd Qu.	Max.
Analog	16443	1	3	4	6.31	9	29
Human	78857	1	3	4	6.26	8	29

© 2020 Neugebauer R et al. *JAMA Network Open.*

eTable 26: Distribution of follow-up time (expressed in 90-day intervals) for patients continuously exposed to analog-containing insulin therapy in the primary all-cause mortality analyses (all sites combined).

Follow-up time	Frequency	%	Cumulative Frequency	Cumulative %
[1, 2[	1904	10.06	1904	10.06
[2, 3[	1198	6.33	3102	16.39
[3, 4[	5861	30.96	8963	47.35
[4, 5[	1674	8.84	10637	56.20
[5, 6[	1004	5.30	11641	61.50
[6, 7[	856	4.52	12497	66.02
[7, 8[	760	4.02	13257	70.04
[8, 9[	608	3.21	13865	73.25
[9, 10[	519	2.74	14384	75.99
[10, 11[	457	2.41	14841	78.41
[11, 12[	397	2.10	15238	80.51
[12, 13[	374	1.98	15612	82.48
[13, 17[	1138	6.01	16750	88.49
[17, 21[	904	4.78	17654	93.27
[21, 25[	634	3.35	18288	96.62
[25, 29[	360	1.90	18648	98.52
[29, 33[	197	1.04	18845	99.56
[33, 37[	73	0.39	18918	99.95
$\geq$ 37	10	0.05	18928	100.00
Missing	0	0.00	18928	100.00

© 2020 Neugebauer R et al. *JAMA Network Open.*

eTable 27: Distribution of follow-up time (expressed in 90-day intervals) for patients continuously exposed to human-only insulin therapy in the primary all-cause mortality analyses (all sites combined).

Follow-up time	Frequency	%	Cumulative Frequency	Cumulative %
[1, 2[	8738	8.04	8738	8.04
[2, 3[	6954	6.40	15692	14.44
[3, 4[	34352	31.61	50044	46.05
[4, 5[	10648	9.80	60692	55.85
[5, 6[	6142	5.65	66834	61.50
[6, 7[	5317	4.89	72151	66.39
[7, 8[	4521	4.16	76672	70.55
[8, 9[	3862	3.55	80534	74.11
[9, 10[	3053	2.81	83587	76.92
[10, 11[	2737	2.52	86324	79.44
[11, 12[	2410	2.22	88734	81.65
[12, 13[	2166	1.99	90900	83.65
[13, 17[	5993	5.51	96893	89.16
[17, 21[	4152	3.82	101045	92.98
[21, 25[	2960	2.72	104005	95.71
[25, 29[	2421	2.23	106426	97.93
[29, 33[	1323	1.22	107749	99.15
[33, 37[	818	0.75	108567	99.90
$\geq$ 37	105	0.10	108672	100.00
Missing	0	0.00	108672	100.00

eTable 28: Summary statistics of the distribution of follow-up time (expressed in 90-day intervals) by exposure regimen in the primary all-cause mortality analyses (all sites combined).

Exposure	N	Min.	1st Qu.	Median	Mean	3rd Qu.	Max.
Analog	18928	1	3	4	7.01	9	37
Human	108672	1	3	4	7.05	9	37

© 2020 Neugebauer R et al. *JAMA Network Open.*

eTable 29: AMI results. The reference exposure regimen is "continuous exposure to human-only insulin therapy".

Analysis	PS estimation	HR	AUC	RD1	RD2
Primary	Crude (no weight)	1.1008 [0.9341;1.2675] SE=0.0851, p=0.236	0.372	0.001 [-9e-04;0.0029] SE=0.001, p=0.301	5e-04 [-0.0025;0.0036] SE=0.0015, p=0.726
all sites, 180-day	Logistic 1	1.2153 [0.9794;1.4512] SE=0.1203, p=0.074	0.086	0.0019 [-8e-04;0.0045] SE=0.0014, p=0.176	0.0035 [-0.0011;0.008] SE=0.0023, p=0.14
prescription duration	Logistic 2	1.079 [0.8579;1.3] SE=0.1128, p=0.484	0.594	4e-04 [-0.0021;0.0029] SE=0.0013, p=0.726	8e-04 [-0.0035;0.0052] SE=0.0022, p=0.705
	Logistic 3	1.1052 [0.7639;1.4464] SE=0.1741, p=0.546	0.889	-0.0014 [-0.0039;0.0011] SE=0.0013, p=0.26	-5e-04 [-0.0055;0.0045] SE=0.0026, p=0.853
	SL	1.1085 [0.7665;1.4505] SE=0.1745, p=0.534	0.87	-0.0015 [-0.004;0.001] SE=0.0013, p=0.252	-4e-04 [-0.0054;0.0046] SE=0.0026, p=0.889
Sensitivity 1	Crude (no weight)	1.065 [0.9208;1.2092] SE=0.0736, p=0.377	0.498	7e-04 [-0.001;0.0023] SE=8e-04, p=0.41	4e-04 [-0.0023;0.003] SE=0.0013, p=0.793
all sites, 365-day	Logistic 1	1.1298 [0.9373;1.3222] SE=0.0982, p=0.186	0.27	0.0014 [-9e-04;0.0038] SE=0.0012, p=0.226	0.0016 [-0.0019;0.0052] SE=0.0018, p=0.368
prescription duration	Logistic 2	1.0111 [0.8319;1.1902] SE=0.0914, p=0.904	0.81	2e-04 [-0.002;0.0024] SE=0.0011, p=0.842	-9e-04 [-0.0042;0.0025] SE=0.0017, p=0.62
	Logistic 3	1.0336 [0.7842;1.2829] SE=0.1272, p=0.792	0.892	-6e-04 [-0.0031;0.0019] SE=0.0013, p=0.637	-0.0012 [-0.0052;0.0028] SE=0.002, p=0.563
	SL	1.0325 [0.7829;1.2821] SE=0.1274, p=0.798	0.892	-6e-04 [-0.0031;0.0019] SE=0.0013, p=0.625	-0.0011 [-0.0051;0.0029] SE=0.002, p=0.58
Sensitivity 2 Single site with	Crude (no weight)	1.1665 [0.9264;1.4065] SE=0.1225, p=0.174	0.411	0.0012 [-0.0011;0.0036] SE=0.0012, p=0.312	5e-04 [-0.0033;0.0042] SE=0.0019, p=0.809
most variability in baseline	Logistic 1	1.3087 [0.9737;1.6437] SE=0.1709, p=0.071	0.18	0.0023 [-9e-04;0.0056] SE=0.0017, p=0.157	0.0015 [-0.0032;0.0063] SE=0.0024, p=0.528
insulin therapy over time,	Logistic 2	1.1865 [0.865;1.5079] SE=0.164, p=0.256	0.538	0.0014 [-0.0018;0.0046] SE=0.0016, p=0.386	-2e-04 [-0.0052;0.0048] SE=0.0026, p=0.938
180-day prescription	Logistic 3	1.1074 [0.7554;1.4594] SE=0.1796, p=0.55	0.895	1e-04 [-0.0031;0.0032] SE=0.0016, p=0.974	-0.0016 [-0.0069;0.0038] SE=0.0027, p=0.568
duration	SL	1.1063 [0.7563;1.4563] SE=0.1786, p=0.552	0.888	0 [-0.0031;0.0032] SE=0.0016, p=0.981	-0.0016 [-0.0068;0.0037] SE=0.0027, p=0.564
Sensitivity 3	Crude (no weight)	1.123 [0.9439;1.302] SE=0.0914, p=0.178	0.281	0.0011 [-9e-04;0.0032] SE=0.0011, p=0.278	0.0011 [-0.0022;0.0044] SE=0.0017, p=0.505
Site favoring analog-containing	Logistic 1	1.2624 [1.0223;1.5025] SE=0.1225, p=0.032	0.046	0.0022 [-5e-04;0.0048] SE=0.0014, p=0.111	0.0039 [-7e-04;0.0086] SE=0.0024, p=0.098
insulin at index excluded,	Logistic 2	1.1208 [0.8941;1.3474] SE=0.1157, p=0.296	0.398	8e-04 [-0.0017;0.0033] SE=0.0013, p=0.534	0.0014 [-0.0031;0.0058] SE=0.0023, p=0.551
180-day prescription	Logistic 3	1.1572 [0.7908;1.5236] SE=0.1869, p=0.4	0.701	-0.0011 [-0.0036;0.0014] SE=0.0013, p=0.374	-1e-04 [-0.0051;0.005] SE=0.0026, p=0.977
duration	SL	1.1607 [0.7907;1.5306] SE=0.1888, p=0.395	0.687	-0.0012 [-0.0036;0.0013] SE=0.0013, p=0.362	0 [-0.005;0.0051] SE=0.0026, p=0.996

© 2020 Neugebauer R et al. *JAMA Network Open.*

eTable 30: CHF results. The reference exposure regimen is “continuous exposure to human-only insulin therapy”.

Analysis	PS estimation	HR	AUC	RD1	RD2
Primary	Crude (no weight)	1.1052 [0.9851;1.2252]<br>SE=0.0612, p=0.086	0.132	0.0016 [-0.001;0.0041]<br>SE=0.0013, p=0.224	0.0012 [-0.0027;0.0051]<br>SE=0.002, p=0.538
all sites,	Logistic 1	1.0704 [0.9226;1.2182]<br>SE=0.0754, p=0.351	0.634	0.0011 [-0.0023;0.0046]<br>SE=0.0018, p=0.513	-0.0016 [-0.0068;0.0036]<br>SE=0.0026, p=0.548
180-day prescription duration	Logistic 2	0.9763 [0.8336;1.119]<br>SE=0.0728, p=0.745	0.469	-0.0011 [-0.0045;0.0023]<br>SE=0.0017, p=0.521	-0.0042 [-0.0092;9e-04]<br>SE=0.0026, p=0.107
	Logistic 3	0.9343 [0.7492;1.1193]<br>SE=0.0944, p=0.486	0.346	-0.0019 [-0.0063;0.0026]<br>SE=0.0023, p=0.412	-0.005 [-0.0112;0.0013]<br>SE=0.0032, p=0.122
	SL	0.9305 [0.7462;1.1149]<br>SE=0.094, p=0.46	0.319	-0.002 [-0.0064;0.0025]<br>SE=0.0023, p=0.389	-0.0052 [-0.0114;0.0011]<br>SE=0.0032, p=0.106
Sensitivity 1	Crude (no weight)	1.1185 [1.0092;1.2278]<br>SE=0.0558, p=0.034	0.023	0.0024 [0;0.0048]<br>SE=0.0012, p=0.046	0.0029 [-6e-04;0.0064]<br>SE=0.0018, p=0.103
all sites,	Logistic 1	1.1074 [0.9677;1.2471]<br>SE=0.0713, p=0.132	0.112	0.0032 [-2e-04;0.0066]<br>SE=0.0017, p=0.066	0.0024 [-0.0025;0.0074]<br>SE=0.0025, p=0.338
365-day prescription duration	Logistic 2	1.0111 [0.8778;1.1443]<br>SE=0.068, p=0.871	0.908	7e-04 [-0.0026;0.0039]<br>SE=0.0017, p=0.691	-6e-04 [-0.0052;0.004]<br>SE=0.0023, p=0.803
	Logistic 3	0.957 [0.7807;1.1332]<br>SE=0.0899, p=0.632	0.594	-1e-04 [-0.0047;0.0045]<br>SE=0.0024, p=0.965	-0.0023 [-0.0081;0.0035]<br>SE=0.003, p=0.429
	SL	0.9531 [0.7778;1.1284]<br>SE=0.0894, p=0.6	0.563	-1e-04 [-0.0048;0.0045]<br>SE=0.0024, p=0.95	-0.0025 [-0.0083;0.0033]<br>SE=0.003, p=0.393
Sensitivity 2<br>Single site with	Crude (no weight)	1.007 [0.8637;1.1504]<br>SE=0.0731, p=0.923	0.798	8e-04 [-0.0027;0.0042]<br>SE=0.0018, p=0.66	-0.0043 [-0.0092;7e-04]<br>SE=0.0025, p=0.091
most variability in baseline	Logistic 1	0.9851 [0.811;1.1593]<br>SE=0.0889, p=0.867	0.353	4e-04 [-0.0042;0.0051]<br>SE=0.0024, p=0.855	-0.0084 [-0.0143;-0.0025]<br>SE=0.003, p=0.005
insulin therapy over time,	Logistic 2	0.886 [0.7247;1.0473]<br>SE=0.0823, p=0.166	0.035	-0.0026 [-0.0071;0.0019]<br>SE=0.0023, p=0.264	-0.0109 [-0.0166;-0.0051]<br>SE=0.0029, p=0
180-day prescription	Logistic 3	0.8303 [0.6557;1.0049]<br>SE=0.0891, p=0.057	0.019	-0.0031 [-0.0084;0.0022]<br>SE=0.0027, p=0.247	-0.0118 [-0.0182;-0.0054]<br>SE=0.0033, p=0
duration	SL	0.8245 [0.6518;0.9971]<br>SE=0.0881, p=0.046	0.014	-0.0033 [-0.0085;0.0019]<br>SE=0.0027, p=0.216	-0.0121 [-0.0185;-0.0057]<br>SE=0.0033, p=0
Sensitivity 3	Crude (no weight)	1.1275 [0.9986;1.2564]<br>SE=0.0658, p=0.053	0.093	0.0024 [-3e-04;0.0052]<br>SE=0.0014, p=0.086	9e-04 [-0.0031;0.005]<br>SE=0.0021, p=0.658
Site favoring analog-containing	Logistic 1	1.0865 [0.936;1.2371]<br>SE=0.0768, p=0.26	0.517	0.0014 [-0.0021;0.0048]<br>SE=0.0018, p=0.444	-0.0013 [-0.0065;0.0039]<br>SE=0.0026, p=0.616
insulin at index excluded,	Logistic 2	0.9871 [0.842;1.1322]<br>SE=0.074, p=0.862	0.562	-8e-04 [-0.0043;0.0026]<br>SE=0.0017, p=0.628	-0.0042 [-0.0092;8e-04]<br>SE=0.0025, p=0.097
180-day prescription	Logistic 3	0.9371 [0.7417;1.1324]<br>SE=0.0997, p=0.528	0.358	-0.0016 [-0.0062;0.0031]<br>SE=0.0024, p=0.512	-0.0056 [-0.012;8e-04]<br>SE=0.0032, p=0.084
duration	SL	0.9333 [0.7388;1.1278]<br>SE=0.0992, p=0.502	0.333	-0.0016 [-0.0063;0.003]<br>SE=0.0024, p=0.487	-0.0058 [-0.0121;6e-04]<br>SE=0.0032, p=0.074

© 2020 Neugebauer R et al. *JAMA Network Open.*

eTable 31: CVA results. The reference exposure regimen is “continuous exposure to human-only insulin therapy”.

Analysis	PS estimation	HR	AUC	RD1	RD2
Primary	Crude (no weight)	1.2202 [1.0205;1.4198] SE=0.1019, p=0.031	0.023	0.0016 [-2e-04;0.0033] SE=9e-04, p=0.085	0.0031 [3e-04;0.0059] SE=0.0014, p=0.029
all sites, 180-day	Logistic 1	1.1912 [0.9291;1.4534] SE=0.1337, p=0.153	0.134	0.0019 [-6e-04;0.0045] SE=0.0013, p=0.135	0.0027 [-0.0011;0.0065] SE=0.0019, p=0.171
prescription duration	Logistic 2	1.0725 [0.8248;1.3202] SE=0.1264, p=0.566	0.533	8e-04 [-0.0016;0.0031] SE=0.0012, p=0.514	0.0017 [-0.0022;0.0055] SE=0.002, p=0.402
	Logistic 3	1.3021 [0.8133;1.791] SE=0.2494, p=0.226	0.241	0.0035 [-0.0024;0.0094] SE=0.003, p=0.244	0.0044 [-0.0026;0.0115] SE=0.0036, p=0.218
	SL	1.2969 [0.8112;1.7826] SE=0.2478, p=0.231	0.247	0.0035 [-0.0024;0.0093] SE=0.003, p=0.246	0.0043 [-0.0027;0.0113] SE=0.0036, p=0.229
Sensitivity 1	Crude (no weight)	1.1366 [0.9697;1.3034] SE=0.0851, p=0.109	0.093	0.001 [-6e-04;0.0025] SE=8e-04, p=0.22	0.0021 [-4e-04;0.0045] SE=0.0012, p=0.095
all sites, 365-day	Logistic 1	1.1129 [0.8915;1.3343] SE=0.113, p=0.317	0.207	0.0017 [-6e-04;0.0041] SE=0.0012, p=0.143	0.0019 [-0.0015;0.0052] SE=0.0017, p=0.274
prescription duration	Logistic 2	1.0315 [0.8194;1.2436] SE=0.1082, p=0.771	0.607	8e-04 [-0.0014;0.003] SE=0.0011, p=0.49	0.0011 [-0.0023;0.0044] SE=0.0017, p=0.531
	Logistic 3	1.1723 [0.8005;1.5442] SE=0.1897, p=0.364	0.32	0.0024 [-0.0017;0.0066] SE=0.0021, p=0.252	0.0027 [-0.0025;0.0078] SE=0.0026, p=0.307
	SL	1.1653 [0.7957;1.5349] SE=0.1886, p=0.381	0.334	0.0024 [-0.0017;0.0065] SE=0.0021, p=0.257	0.0026 [-0.0026;0.0077] SE=0.0026, p=0.328
Sensitivity 2 Single site with	Crude (no weight)	1.17 [0.9231;1.4169] SE=0.126, p=0.177	0.109	0.0013 [-0.0011;0.0037] SE=0.0012, p=0.292	0.0028 [-9e-04;0.0066] SE=0.0019, p=0.141
most variability in baseline	Logistic 1	1.103 [0.8144;1.3916] SE=0.1473, p=0.484	0.297	0.0016 [-0.0014;0.0045] SE=0.0015, p=0.292	0.0025 [-0.0021;0.007] SE=0.0023, p=0.29
insulin therapy over time,	Logistic 2	1.0336 [0.7526;1.3145] SE=0.1433, p=0.815	0.624	0.001 [-0.002;0.0039] SE=0.0015, p=0.514	0.0016 [-0.0033;0.0065] SE=0.0025, p=0.53
180-day prescription	Logistic 3	1.1008 [0.5548;1.6467] SE=0.2785, p=0.718	0.659	0.0023 [-0.0055;0.0102] SE=0.004, p=0.562	0.0023 [-0.0067;0.0113] SE=0.0046, p=0.623
duration	SL	1.1019 [0.556;1.6477] SE=0.2785, p=0.715	0.658	0.0023 [-0.0055;0.0102] SE=0.004, p=0.559	0.0022 [-0.0068;0.0112] SE=0.0046, p=0.63
Sensitivity 3	Crude (no weight)	1.2275 [1.0149;1.4401] SE=0.1085, p=0.036	0.021	0.0017 [-2e-04;0.0036] SE=0.001, p=0.084	0.0035 [5e-04;0.0066] SE=0.0015, p=0.022
Site favoring analog-containing	Logistic 1	1.2056 [0.9375;1.4737] SE=0.1368, p=0.133	0.11	0.0021 [-5e-04;0.0046] SE=0.0013, p=0.109	0.0029 [-0.001;0.0068] SE=0.002, p=0.144
insulin at index excluded,	Logistic 2	1.0833 [0.8297;1.3369] SE=0.1294, p=0.52	0.463	0.001 [-0.0014;0.0033] SE=0.0012, p=0.425	0.0019 [-0.002;0.0059] SE=0.002, p=0.345
180-day prescription	Logistic 3	1.3351 [0.8119;1.8583] SE=0.2669, p=0.209	0.215	0.0039 [-0.0023;0.01] SE=0.0031, p=0.215	0.0049 [-0.0024;0.0122] SE=0.0037, p=0.192
duration	SL	1.3324 [0.8117;1.8531] SE=0.2657, p=0.211	0.219	0.0039 [-0.0023;0.01] SE=0.0031, p=0.216	0.0048 [-0.0025;0.0121] SE=0.0037, p=0.2

© 2020 Neugebauer R et al. *JAMA Network Open.*

eTable 32: CVD mortality results. The reference exposure regimen is "continuous exposure to human-only insulin therapy".

Analysis	PS estimation	HR	AUC	RD1	RD2
Primary	Crude (no weight)	0.9666 [0.8277;1.1054]<br>SE=0.0708, p=0.637	0.438	4e-04 [-0.002;0.0029]<br>SE=0.0012, p=0.716	-0.0032 [-0.0067;2e-04]<br>SE=0.0018, p=0.069
all sites,<br>180-day	Logistic 1	1.1019 [0.9065;1.2972]<br>SE=0.0997, p=0.307	0.569	0.0025 [-0.0014;0.0063]<br>SE=0.002, p=0.205	-0.0015 [-0.007;0.004]<br>SE=0.0028, p=0.592
prescription<br>duration	Logistic 2	0.9773 [0.7948;1.1597]<br>SE=0.0931, p=0.807	0.633	9e-04 [-0.003;0.0047]<br>SE=0.002, p=0.666	-0.0039 [-0.01;0.0023]<br>SE=0.0031, p=0.216
	Logistic 3	1.2624 [0.8637;1.6611]<br>SE=0.2034, p=0.197	0.22	0.0095 [-6e-04;0.0196]<br>SE=0.0052, p=0.066	0.0035 [-0.0074;0.0145]<br>SE=0.0056, p=0.528
	SL	1.2599 [0.8619;1.6578]<br>SE=0.2031, p=0.201	0.226	0.0094 [-7e-04;0.0195]<br>SE=0.0052, p=0.067	0.0034 [-0.0075;0.0144]<br>SE=0.0056, p=0.539
Sensitivity 1	Crude (no weight)	0.9503 [0.8289;1.0717]<br>SE=0.062, p=0.422	0.445	1e-04 [-0.0021;0.0024]<br>SE=0.0011, p=0.897	-0.0022 [-0.0054;0.001]<br>SE=0.0016, p=0.185
all sites,<br>365-day	Logistic 1	1.0811 [0.905;1.2572]<br>SE=0.0899, p=0.367	0.343	0.0026 [-9e-04;0.0061]<br>SE=0.0018, p=0.152	0.0016 [-0.0039;0.0071]<br>SE=0.0028, p=0.561
prescription<br>duration	Logistic 2	0.9579 [0.7803;1.1356]<br>SE=0.0906, p=0.642	0.726	4e-04 [-0.003;0.0038]<br>SE=0.0017, p=0.817	-0.0021 [-0.0087;0.0045]<br>SE=0.0034, p=0.53
	Logistic 3	1.202 [0.8787;1.5253]<br>SE=0.1649, p=0.221	0.188	0.0061 [-7e-04;0.0129]<br>SE=0.0035, p=0.078	0.0047 [-0.0055;0.0148]<br>SE=0.0052, p=0.368
	SL	1.1972 [0.8789;1.5155]<br>SE=0.1624, p=0.225	0.193	0.0061 [-7e-04;0.0129]<br>SE=0.0035, p=0.077	0.0044 [-0.0053;0.0142]<br>SE=0.005, p=0.374
Sensitivity 2<br>Single site with	Crude (no weight)	0.8253 [0.6675;0.9831]<br>SE=0.0805, p=0.03	0.011	-0.0022 [-0.0051;8e-04]<br>SE=0.0015, p=0.15	-0.0075 [-0.0121;-0.003]<br>SE=0.0023, p=0.001
most variability<br>in baseline	Logistic 1	0.9277 [0.7139;1.1416]<br>SE=0.1091, p=0.508	0.18	-0.001 [-0.005;0.0031]<br>SE=0.0021, p=0.646	-0.0074 [-0.0138;-0.001]<br>SE=0.0033, p=0.024
insulin therapy<br>over time,	Logistic 2	0.8454 [0.6397;1.051]<br>SE=0.1049, p=0.141	0.108	-8e-04 [-0.0058;0.0042]<br>SE=0.0026, p=0.748	-0.0089 [-0.0162;-0.0015]<br>SE=0.0037, p=0.018
180-day<br>prescription	Logistic 3	0.9773 [0.5834;1.3711]<br>SE=0.2009, p=0.91	0.835	0.0039 [-0.0071;0.0148]<br>SE=0.0056, p=0.489	-0.0054 [-0.0171;0.0064]<br>SE=0.006, p=0.369
duration	SL	0.9675 [0.5808;1.3543]<br>SE=0.1973, p=0.869	0.782	0.0035 [-0.0071;0.0142]<br>SE=0.0055, p=0.515	-0.0058 [-0.0173;0.0058]<br>SE=0.0059, p=0.326
Sensitivity 3	Crude (no weight)	0.9734 [0.8265;1.1202]<br>SE=0.0749, p=0.722	0.424	1e-04 [-0.0024;0.0026]<br>SE=0.0013, p=0.913	-0.0033 [-0.007;3e-04]<br>SE=0.0019, p=0.075
Site favoring<br>analog-containing	Logistic 1	1.0997 [0.9038;1.2955]<br>SE=0.0999, p=0.319	0.58	0.0026 [-0.0013;0.0064]<br>SE=0.002, p=0.186	-0.0016 [-0.007;0.0039]<br>SE=0.0028, p=0.578
insulin at index<br>excluded,	Logistic 2	0.9773 [0.7935;1.1611]<br>SE=0.0938, p=0.808	0.632	0.001 [-0.0028;0.0048]<br>SE=0.0019, p=0.607	-0.0039 [-0.0101;0.0022]<br>SE=0.0031, p=0.213
180-day<br>prescription	Logistic 3	1.2789 [0.8533;1.7045]<br>SE=0.2172, p=0.199	0.223	0.0101 [-8e-04;0.0211]<br>SE=0.0056, p=0.07	0.0039 [-0.0079;0.0157]<br>SE=0.006, p=0.515
duration	SL	1.2776 [0.8522;1.703]<br>SE=0.217, p=0.201	0.227	0.0101 [-9e-04;0.021]<br>SE=0.0056, p=0.072	0.0038 [-0.0079;0.0156]<br>SE=0.006, p=0.522

eTable 33: All-cause mortality results. The reference exposure regimen is "continuous exposure to human-only insulin therapy".

Analysis	PS estimation	HR	AUC	RD1	RD2
Primary	Crude (no weight)	1.1411 [1.0478;1.2345] SE=0.0476, p=0.003	0.003	0.0048 [0.0013;0.0082] SE=0.0018, p=0.007	0.0054 [3e-04;0.0105] SE=0.0026, p=0.037
all sites, 180-day	Logistic 1	1.1723 [1.0471;1.2976] SE=0.0639, p=0.007	0.024	0.0058 [9e-04;0.0107] SE=0.0025, p=0.02	0.0038 [-0.003;0.0106] SE=0.0035, p=0.273
prescription duration	Logistic 2	1.0597 [0.9412;1.1782] SE=0.0605, p=0.323	0.477	0.0017 [-0.0032;0.0067] SE=0.0025, p=0.488	0.001 [-0.0071;0.0091] SE=0.0041, p=0.808
	Logistic 3	1.1584 [0.9705;1.3462] SE=0.0958, p=0.098	0.125	0.0083 [-7e-04;0.0172] SE=0.0046, p=0.07	0.006 [-0.0052;0.0172] SE=0.0057, p=0.294
	SL	1.1526 [0.9665;1.3387] SE=0.095, p=0.108	0.142	0.008 [-8e-04;0.0169] SE=0.0045, p=0.076	0.0054 [-0.0056;0.0164] SE=0.0056, p=0.334
Sensitivity 1	Crude (no weight)	1.1411 [1.0593;1.223] SE=0.0418, p=0.001	0	0.0049 [0.0018;0.0081] SE=0.0016, p=0.002	0.0074 [0.0028;0.012] SE=0.0024, p=0.002
all sites, 365-day	Logistic 1	1.1572 [1.0498;1.2646] SE=0.0548, p=0.004	0.002	0.0065 [0.002;0.0109] SE=0.0023, p=0.004	0.0078 [0.0017;0.014] SE=0.0031, p=0.012
prescription duration	Logistic 2	1.0429 [0.9429;1.1428] SE=0.051, p=0.4	0.314	0.0018 [-0.0025;0.0061] SE=0.0022, p=0.419	0.0029 [-0.0035;0.0094] SE=0.0033, p=0.372
	Logistic 3	1.1129 [0.9621;1.2637] SE=0.0769, p=0.142	0.099	0.0064 [-4e-04;0.0132] SE=0.0035, p=0.064	0.0064 [-0.0023;0.0151] SE=0.0044, p=0.15
	SL	1.1085 [0.9589;1.2581] SE=0.0763, p=0.155	0.112	0.0062 [-5e-04;0.013] SE=0.0034, p=0.07	0.006 [-0.0026;0.0146] SE=0.0044, p=0.174
Sensitivity 2 Single site with	Crude (no weight)	1.0387 [0.92;1.1574] SE=0.0606, p=0.522	0.56	8e-04 [-0.0034;0.005] SE=0.0022, p=0.71	0.0016 [-0.0047;0.008] SE=0.0033, p=0.614
most variability in baseline	Logistic 1	1.1595 [0.9941;1.3249] SE=0.0844, p=0.059	0.095	0.0047 [-0.0012;0.0105] SE=0.003, p=0.119	0.0055 [-0.003;0.0139] SE=0.0043, p=0.205
insulin therapy over time,	Logistic 2	1.0693 [0.9082;1.2303] SE=0.0822, p=0.399	0.498	0.0015 [-0.0044;0.0075] SE=0.003, p=0.611	0.0025 [-0.0079;0.0129] SE=0.0053, p=0.641
180-day prescription	Logistic 3	1.1853 [0.9164;1.4542] SE=0.1372, p=0.177	0.218	0.0082 [-0.0042;0.0206] SE=0.0063, p=0.196	0.0075 [-0.0085;0.0235] SE=0.0082, p=0.361
duration	SL	1.1829 [0.916;1.4499] SE=0.1362, p=0.179	0.225	0.0082 [-0.0043;0.0206] SE=0.0063, p=0.198	0.0073 [-0.0083;0.0229] SE=0.008, p=0.362
Sensitivity 3	Crude (no weight)	1.0909 [0.9943;1.1875] SE=0.0493, p=0.065	0.105	0.0032 [-4e-04;0.0067] SE=0.0018, p=0.08	0.0022 [-0.003;0.0075] SE=0.0027, p=0.401
Site favoring analog-containing	Logistic 1	1.1829 [1.0561;1.3098] SE=0.0647, p=0.005	0.018	0.006 [0.0012;0.0109] SE=0.0025, p=0.015	0.004 [-0.0028;0.0107] SE=0.0035, p=0.251
insulin at index excluded,	Logistic 2	1.0672 [0.9471;1.1872] SE=0.0612, p=0.273	0.44	0.0018 [-0.003;0.0067] SE=0.0025, p=0.455	0.0012 [-0.0069;0.0093] SE=0.0041, p=0.776
180-day prescription	Logistic 3	1.1584 [0.963;1.3537] SE=0.0997, p=0.112	0.147	0.0082 [-0.0011;0.0176] SE=0.0048, p=0.084	0.0059 [-0.0058;0.0177] SE=0.006, p=0.321
duration	SL	1.1514 [0.9583;1.3445] SE=0.0985, p=0.124	0.167	0.0079 [-0.0013;0.0171] SE=0.0047, p=0.093	0.0053 [-0.0062;0.0167] SE=0.0058, p=0.368

eTable 34: Summary statistics of the inverse probability weights (IPW) involved in the AMI analyses.

Analysis	PS estimation	99th percentile	99.99^{th} percentile	Maximum	Percentage of IPW ≥ 20
Primary	Logistic 1	2.88	8.51	226.08	0.02
	Logistic 2	4.77	24.51	793182.22	0.12
	Logistic 3	5.27	26.53	403173.85	0.15
	SL	4.95	23.36	206848.68	0.12
Sensitivity 1 (365)	Logistic 1	2.99	9.42	341.97	0.03
	Logistic 2	4.94	22.43	2564.02	0.12
	Logistic 3	5.54	25.23	2225.97	0.14
	SL	5.12	21.77	1962.56	0.11
Sensitivity 2 (single site)	Logistic 1	3.09	6.77	104.88	0.01
	Logistic 2	4.78	24.40	104729.23	0.12
	Logistic 3	5.42	29.01	99615.08	0.16
	SL	5.04	24.99	73964.02	0.13
Sensitivity 3 (one site excluded)	Logistic 1	2.84	7.27	202.45	0.01
	Logistic 2	4.70	22.37	859583.30	0.11
	Logistic 3	5.24	24.26	430850.17	0.13
	SL	4.91	21.37	230787.13	0.11

© 2020 Neugebauer R et al. *JAMA Network Open.*

eTable 35: Summary statistics of the inverse probability weights (IPW) involved in the CHF analyses.

Analysis	PS estimation	99th percentile	99.99^{th} percentile	Maximum	Percentage of IPW $\geq$ 20
Primary	Logistic 1	2.76	7.48	182.58	0.02
	Logistic 2	4.67	20.10	10817.68	0.10
	Logistic 3	5.16	22.75	10136.96	0.13
	SL	4.80	20.38	9646.87	0.10
Sensitivity 1 (365)	Logistic 1	2.84	8.27	179.49	0.03
	Logistic 2	4.85	21.48	3092.48	0.11
	Logistic 3	5.42	24.15	3474.22	0.13
	SL	5.01	20.98	3149.91	0.11
Sensitivity 2 (single site)	Logistic 1	2.94	6.15	96.09	0.00
	Logistic 2	4.78	19.63	545.68	0.10
	Logistic 3	5.26	24.40	438.06	0.13
	SL	4.92	20.77	246.44	0.11
Sensitivity 3 (one site excluded)	Logistic 1	2.72	6.48	210.50	0.01
	Logistic 2	4.60	18.52	11734.83	0.09
	Logistic 3	5.10	21.13	11313.56	0.11
	SL	4.77	18.68	10696.20	0.09

© 2020 Neugebauer R et al. *JAMA Network Open.*

eTable 36: Summary statistics of the inverse probability weights (IPW) involved in the CVA analyses.

Analysis	PS estimation	99th percentile	99.99^{th} percentile	Maximum	Percentage of IPW ≥ 20
Primary	Logistic 1	2.92	8.34	231.74	0.03
	Logistic 2	4.80	24.68	790421.98	0.13
	Logistic 3	5.29	27.03	404390.61	0.15
	SL	4.95	23.65	200042.19	0.12
Sensitivity 1 (365)	Logistic 1	3.01	9.28	354.40	0.03
	Logistic 2	5.00	23.10	25531.32	0.12
	Logistic 3	5.59	25.63	19474.62	0.14
	SL	5.17	22.00	15920.99	0.12
Sensitivity 2 (single site)	Logistic 1	3.11	6.80	189.65	0.00
	Logistic 2	4.86	25.04	118032.18	0.13
	Logistic 3	5.47	29.97	114610.75	0.17
	SL	5.09	25.66	92110.35	0.14
Sensitivity 3 (one site excluded)	Logistic 1	2.87	7.07	310.48	0.01
	Logistic 2	4.73	22.54	860883.03	0.11
	Logistic 3	5.25	24.49	434861.23	0.13
	SL	4.91	21.44	229026.02	0.11

© 2020 Neugebauer R et al. *JAMA Network Open.*

eTable 37: Summary statistics of the inverse probability weights (IPW) involved in the CVD mortality analyses.

Analysis	PS estimation	99th percentile	99.99^{th} percentile	Maximum	Percentage of IPW ≥ 20
Primary	Logistic 1	2.53	6.86	58.05	0.01
	Logistic 2	4.58	20.70	1004.69	0.10
	Logistic 3	5.25	23.90	825.98	0.13
	SL	4.95	20.76	569.44	0.11
Sensitivity 1 (365)	Logistic 1	2.74	7.98	73.11	0.02
	Logistic 2	5.04	20.18	1401.33	0.10
	Logistic 3	5.85	23.19	1524.98	0.12
	SL	5.49	20.49	1199.83	0.10
Sensitivity 2 (single site)	Logistic 1	2.55	4.87	17.91	0.00
	Logistic 2	4.39	20.58	924.25	0.10
	Logistic 3	5.35	23.97	882.96	0.12
	SL	5.04	20.56	459.27	0.10
Sensitivity 3 (one site excluded)	Logistic 1	2.46	5.47	25.79	0.00
	Logistic 2	4.50	18.98	992.26	0.09
	Logistic 3	5.18	21.87	846.68	0.12
	SL	4.87	19.38	588.49	0.09

© 2020 Neugebauer R et al. *JAMA Network Open.*

eTable 38: Summary statistics of the inverse probability weights (IPW) involved in the all-cause mortality analyses.

Analysis	PS estimation	99th percentile	99.99^{th} percentile	Maximum	Percentage of IPW ≥ 20
Primary	Logistic 1	2.48	5.72	65.49	0.01
	Logistic 2	4.09	16.80	1116.62	0.08
	Logistic 3	4.65	19.92	1934.06	0.10
	SL	4.33	17.31	1374.05	0.08
Sensitivity 1 (365)	Logistic 1	2.48	5.88	72.65	0.01
	Logistic 2	4.30	17.30	466.03	0.08
	Logistic 3	4.95	20.21	729.87	0.10
	SL	4.56	17.21	709.81	0.08
Sensitivity 2 (single site)	Logistic 1	2.69	5.11	18.38	0.00
	Logistic 2	4.32	16.74	398.01	0.08
	Logistic 3	4.98	22.20	405.55	0.11
	SL	4.64	18.30	166.43	0.09
Sensitivity 3 (one site excluded)	Logistic 1	2.42	4.87	52.95	0.00
	Logistic 2	4.05	15.48	965.47	0.07
	Logistic 3	4.58	18.50	1713.45	0.09
	SL	4.25	16.00	1240.14	0.07

© 2020 Neugebauer R et al. *JAMA Network Open.*

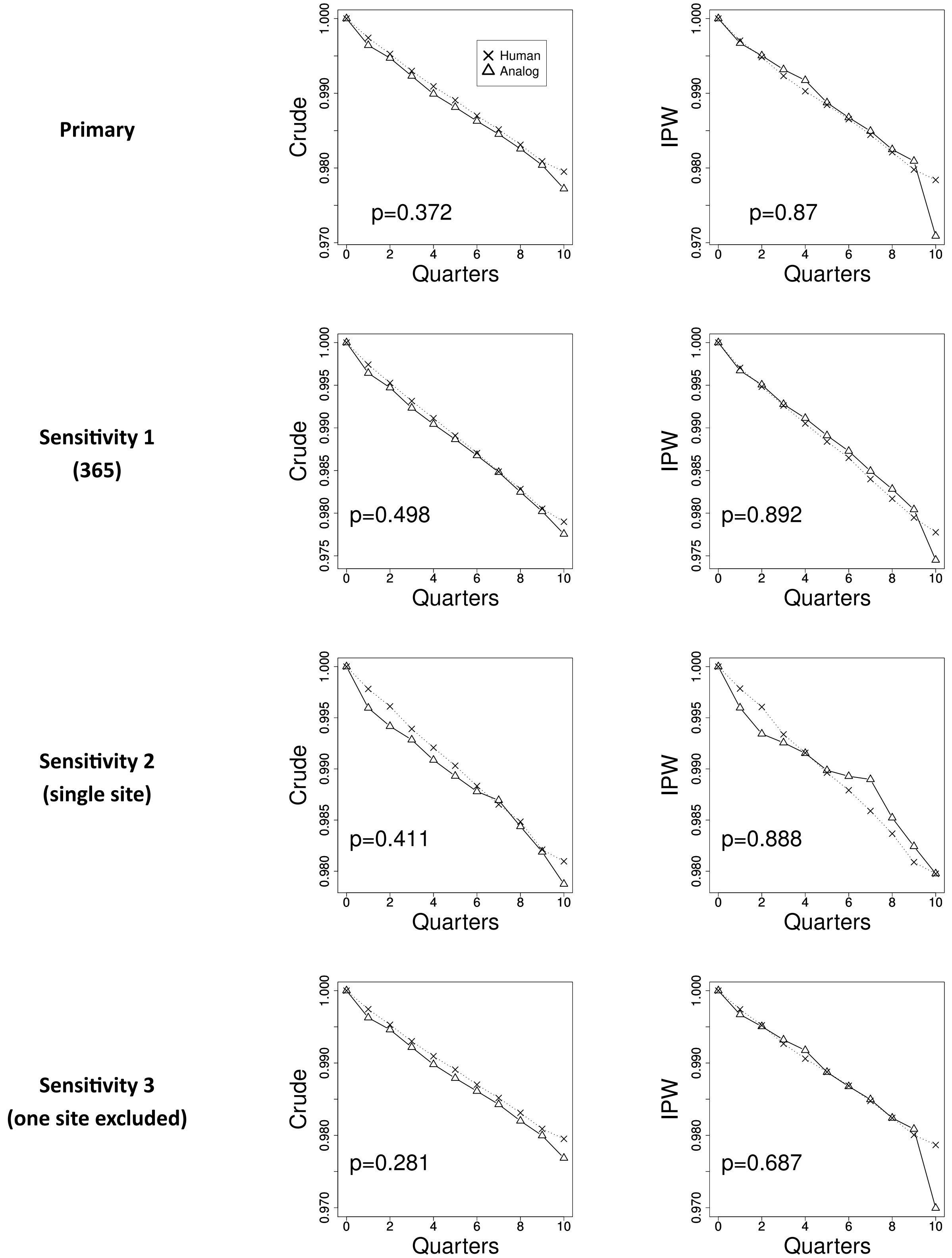

eFigure 1: Survival curve estimates for AMI (primary and sensitivity analyses). The left plots represent the unadjusted estimates. The right plots represent the truncated IPW estimates based on SL estimation of the propensity scores.

© 2020 Neugebauer R et al. *JAMA Network Open.*

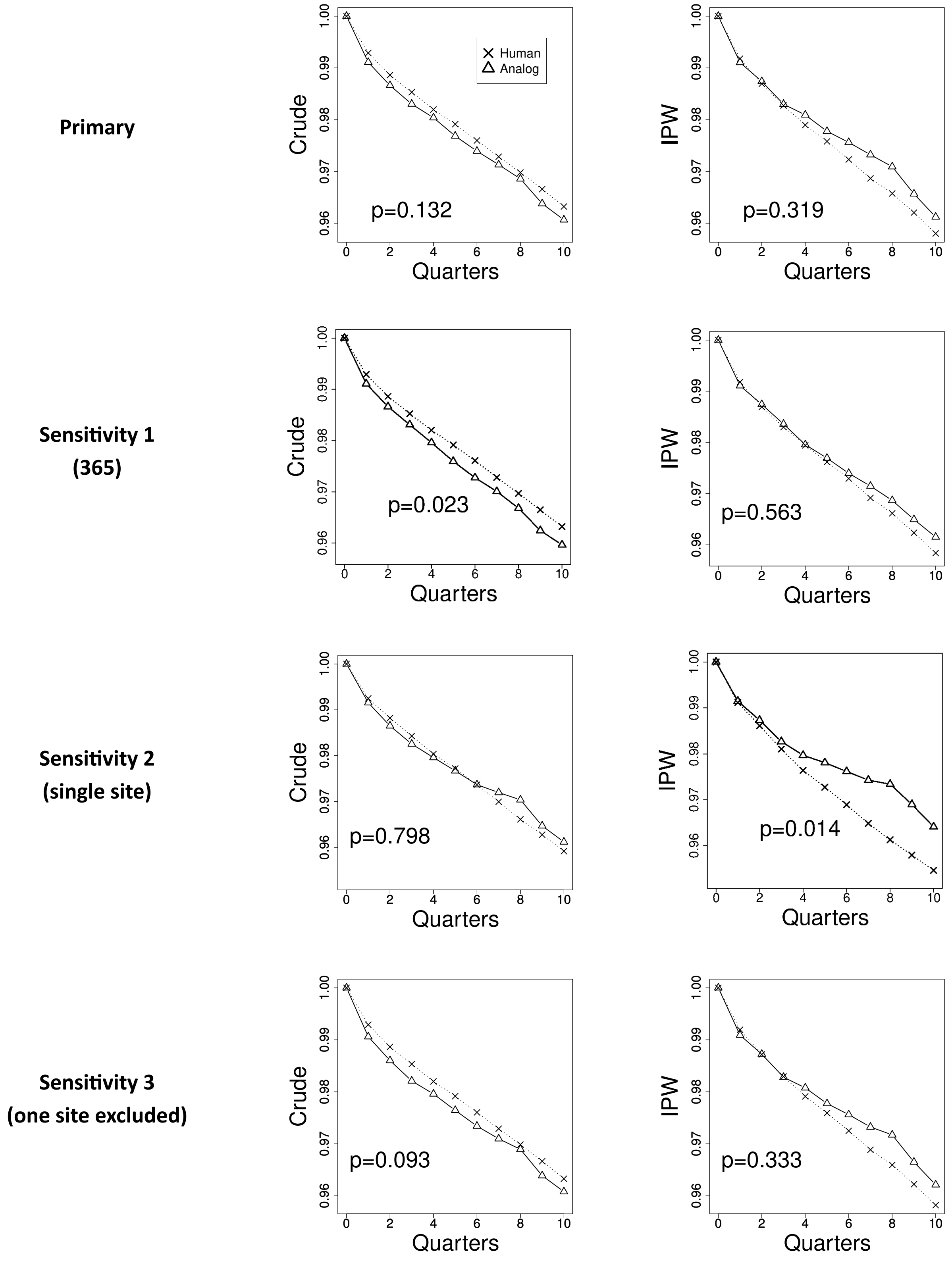

eFigure 2: Survival curve estimates for CHF (primary and sensitivity analyses). The left plots represent the unadjusted estimates. The right plots represent the truncated IPW estimates based on SL estimation of the propensity scores.

© 2020 Neugebauer R et al. *JAMA Network Open.*

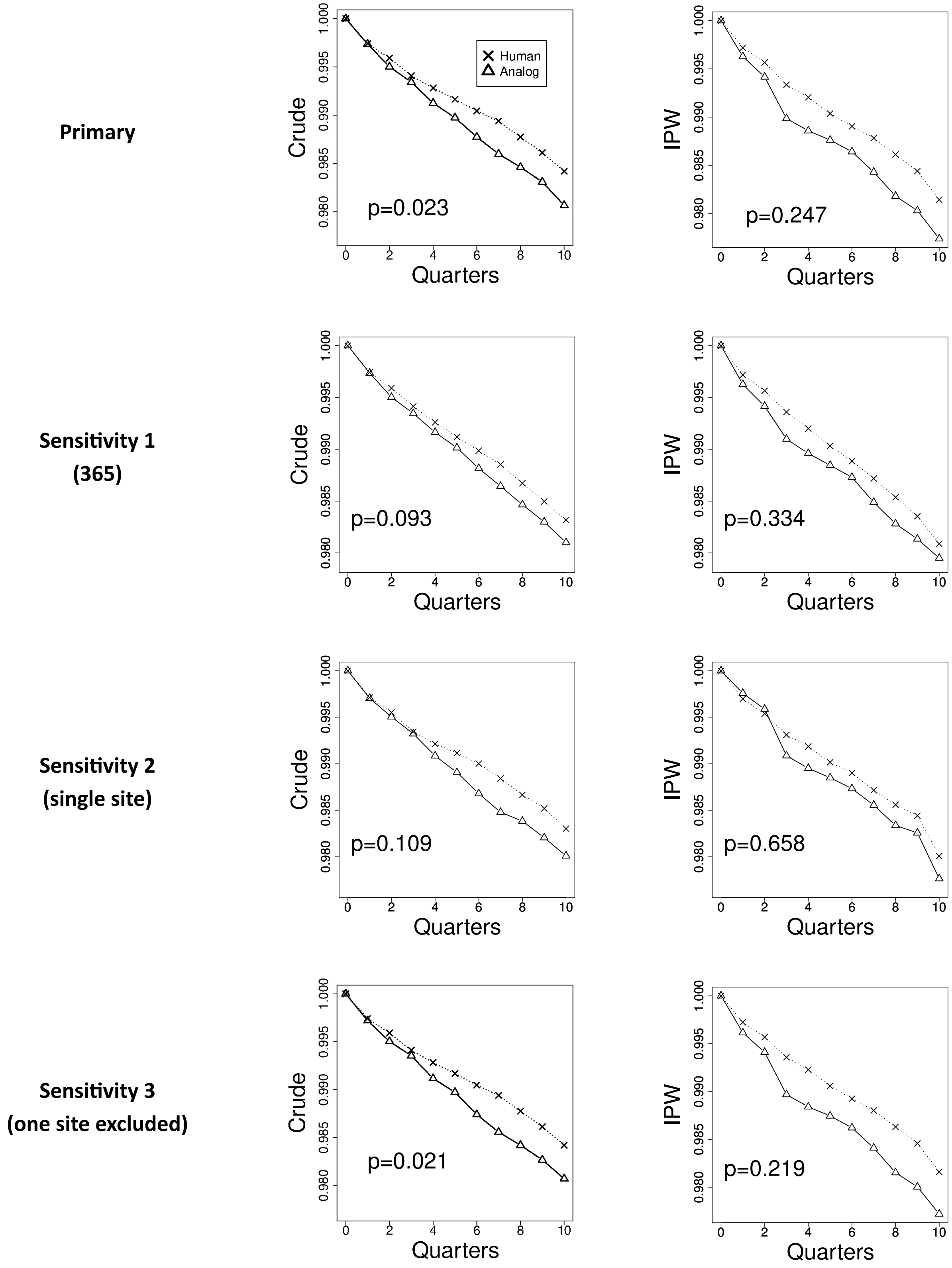

eFigure 3: Survival curve estimates for CVA (primary and sensitivity analyses). The left plots represent the unadjusted estimates. The right plots represent the truncated IPW estimates based on SL estimation of the propensity scores.

© 2020 Neugebauer R et al. *JAMA Network Open.*

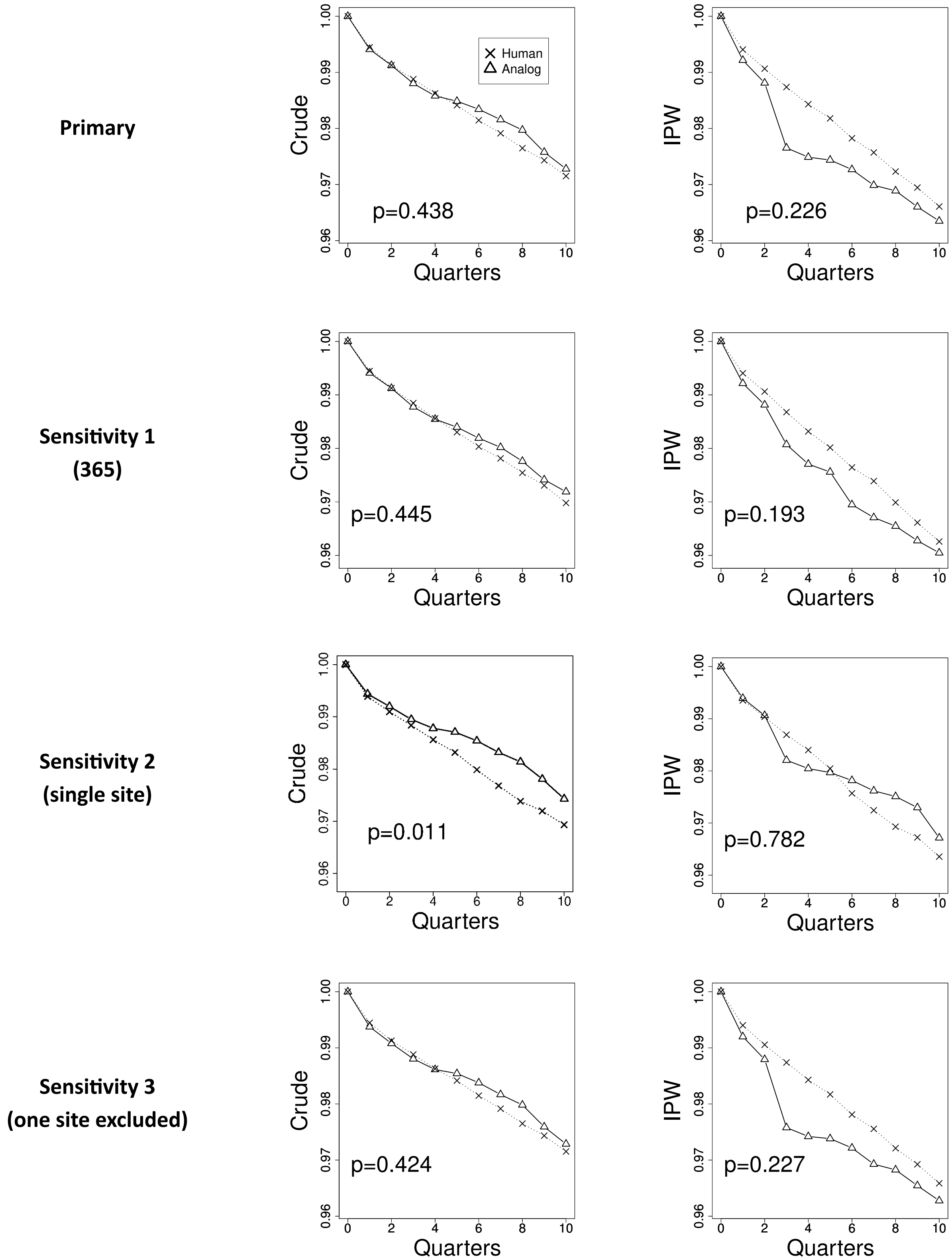

eFigure 4: Survival curve estimates for CVD mortality (primary and sensitivity analyses). The left plots represent the unadjusted estimates. The right plots represent the truncated IPW estimates based on SL estimation of the propensity scores.

© 2020 Neugebauer R et al. *JAMA Network Open.*

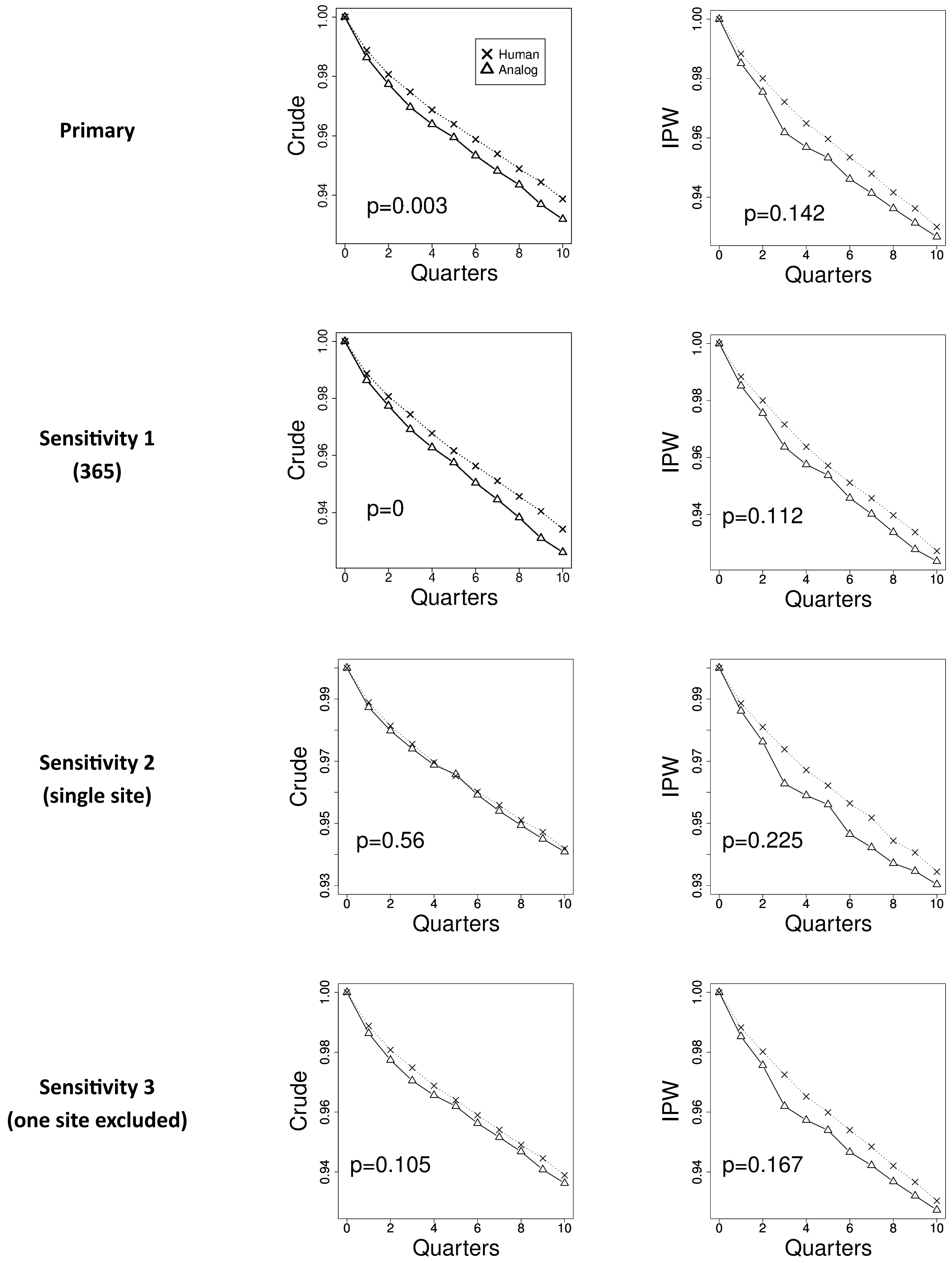

eFigure 5: Survival curve estimates for all-cause mortality (primary and sensitivity analyses). The left plots represent the unadjusted estimates. The right plots represent the truncated IPW estimates based on SL estimation of the propensity scores.

© 2020 Neugebauer R et al. *JAMA Network Open.*

References Cited

[1] Leong T. K., Tabada G. H., Yang J., Zhu Z., Neugebauer R.. %_MSMstructure SAS macro https://divisionofresearch.kaiserpermanente.org/projects/biostatistics/causalinferencesoftware 2017.

[2] Robins J.M.. Marginal Structural Models in *1997 Proceedings of the American Statistical Association, Section on Bayesian Statistical Science*:1–10 1998.

[3] Neugebauer R., van der Laan M. J.. Nonparametric causal effects based on marginal structural models *Journal of Statistical Planning and Inference.* 2007;137:419 - 434.

[4] van der Laan M. J., Robins J. M.. *Unified methods for censored longitudinal data and causality*. New York: Springer 2003.

[5] Neugebauer R., Schmittdiel J. A., van der Laan M. J.. A Case Study of the Impact of Data-Adaptive Versus Model-Based Estimation of the Propensity Scores on Causal Inferences from Three Inverse Probability Weighting Estimators *Int J Biostat.* 2016;12:131–155.

[6] Polley E.. SuperLearner `https://github.com/ecpolley/SuperLearner` 2011. Version 2.

© 2020 Neugebauer R et al. *JAMA Network Open.*
